# LaeA Controls Virulence and Secondary Metabolism in Apple Canker Pathogen *Valsa mali*

**DOI:** 10.3389/fmicb.2020.581203

**Published:** 2020-11-05

**Authors:** Yaqiong Feng, Zhiyuan Yin, Yuxing Wu, Liangsheng Xu, Hongxia Du, Nana Wang, Lili Huang

**Affiliations:** ^1^State Key Laboratory of Crop Stress Biology for Arid Areas, College of Plant Protection, Northwest A&F University, Yangling, China; ^2^College of Life Science, Northwest A&F University, Yangling, China

**Keywords:** *Valsa mali*, LaeA, virulence, secondary metabolism, transcriptome, proteome, NRPS

## Abstract

Apple Valsa canker is a destructive disease caused by the ascomycete *Valsa mali* and poses a serious threat to apple production. Toxins synthesized by secondary metabolite biosynthetic gene clusters (SMBGCs) have been proven to be crucial for pathogen virulence. A previous study showed that *V. mali* genome contains remarkably expanded SMBGCs and some of their genes were significantly upregulated during infection. In this study, we focus on LaeA, a known regulator of secondary metabolism, for its role in SMBGC regulation, toxin production, and virulence of *V. mali*. Deletion of *VmLaeA* led to greatly reduced virulence with lesion length reduced by 48% on apple twigs. Toxicity tests proved that toxicity of secondary metabolites (SMs) produced by *VmLaeA* deletion mutant (Δ*VmlaeA*) was markedly decreased in comparison with wild-type (WT). Transcriptomic and proteomic analyses of WT and Δ*VmlaeA* indicated that a portion of transporters and about half (31/60) SMBGCs are regulated by *VmLaeA.* Function analysis of eight gene clusters including *PKS7*, *PKS11*, *NRPS14*, *PKS16*, *PKS23*, *PKS31*, *NRPS/PKS33*, and *PKS39* that were differentially expressed at both transcriptional and translational levels showed that four of them (i.e., *PKS11*, *PKS16*, *PKS23*, and *PKS31*) were involved in pigment production and *NRPS14* contributed to virulence. Our findings will provide new insights and gene resources for understanding the role of pathogenicity-related toxins in *V. mali*.

## Introduction

Apple Valsa canker, a destructive disease in apple trees which leads to severe yield losses, is caused by the ascomycete *Valsa mali* (Lee et al., 2006; Li et al., 2013; Wang X. et al., 2014). It was first identified in Japan and is now widespread in eastern Asia. Elucidation of its pathogenesis mechanisms is vital to develop rational, novel control strategies. Pathogenic fungi usually secrete copious amounts of toxins to rapidly kill host cells and establish colonization at the early stage of infection (Doehlemann et al., 2017). Electron microscopy observation of apple bark tissues infected with *V. mali* hyphae found severe tissue maceration and cell necrosis which suggests that toxins may involve in the early stage of infection in *V. mali* (Ke et al., 2013). Toxins, such as the trichothecenes of *Fusarium graminearum* (Kim et al., 2013), T-toxin of *Cochliobolus heterostrophus* (Wu et al., 2012), and tentoxin produced by *Alternaria* spp. (Li et al., 2016), all of which stem from secondary metabolism, are important virulence factors in phytopathogenic fungi. For the causal agent of southern corn leaf blight *C. heterostrophus*, its highly aggressive nature is mostly attributable to its ability to produce the host selective toxin (HST) T-toxin, which causes severe ultrastructural damage to mitochondria in nothing flat (Gregory et al., 1980; Baker et al., 2006). The non-host-specific toxin botrydial, produced by *Botrytis cinerea*, could induce chlorosis and collapse of French bean tissues, and deletion of the botrydial biosynthetic genes would lead to reduced virulence (Deighton et al., 2001; Siewers et al., 2005). Another well-known toxin fumonisin B1 produced by several *Fusarium* spp. can induce host cell death and is necessary for the development of disease symptoms on maize seedlings (Myung et al., 2012). These toxins are all crucial weapons for phytopathogenic fungi and are generally synthesized by secondary metabolite biosynthetic gene clusters (SMBGCs).

Previously, several toxins were isolated from culture filtrates of *V. mali*. They were identified as degradation products of phlorizin, including *p*-hydroxybenzoic acid, *p*-hydroxyacetophenone, phloroglucinol, 3-(*p*-hydroxyphenyl) propionic acid, and protocatechuic acid, and are responsible for the symptoms of Valsa canker (Koganezawa and Sakuma, 1982; Natsume et al., 1982; Wang C. et al., 2014). Five isocoumarins produced by *V. mali* were also shown to promote the infection of *V. mali* on apple trees (Okuno et al., 1986). Remarkably, secondary metabolism-related genes are remarkably expanded in *V. mali* and are significantly upregulated during infection (Ke et al., 2014; Yin et al., 2015). These findings prompt us to speculate that SMBGCs probably play essential roles in *V. mali* infection. However, which SMBGCs are involved in virulence or toxin production is still undefined.

LaeA, a global regulator for secondary metabolism, was first identified in *Aspergillus nidulans* and is now well characterized in many filamentous fungi (Bok and Keller, 2004). LaeA regulates secondary metabolism by forming velvet complexes to induce chromatin modification, which is important for modulation of fungal secondary metabolism (Bayram et al., 2008; Brakhage, 2013). In maize pathogen *C. heterostrophus*, *ChLae1* (an ortholog of *A. nidulans LaeA*) positively regulates *Tox1* expression and T-toxin production (Wu et al., 2012). In *Penicillium expansum*, *LaeA* regulates the patulin gene cluster and concomitant patulin synthesis *in vitro*, other SMBGCs, and secondary metabolism-modulated virulence (Kumar et al., 2017). By performing proteomic analysis of wild-type (WT) and Δ*laeA* mutants in *A. flavus*, enzymes participating in aflatoxin, cyclopiazonic acid, and ustiloxin B biosynthesis were found to be dramatically decreased in the mutant (Lv et al., 2018). Furthermore, *LaeA* is capable of regulating some other infection-related functions in phytopathogenic fungi. For instance, in *lae1* (the deletion mutant of *LaeA* in *B. cinerea*), a set of infection-related proteins was strongly underexpressed, including secreted proteins, carbohydrate-active enzymes (CAZymes), proteases, and transporters except for secondary metabolism-related enzymes (Müller et al., 2018). To date, LaeA is regarded as a regulator of the most common mycotoxins by controlling the expression of secondary metabolism-related genes, including sterigmatocystin (Bok and Keller, 2004), aflatoxin (Yao et al., 2018), fumonisin (Butchko et al., 2012), cyclopiazonic acid (Georgianna et al., 2010), T-toxin (Wu et al., 2012), patulin (Kumar et al., 2017), and ochratoxin (Crespo-Sempere et al., 2013).

Traditionally, toxins are isolated from culture filtrates of pathogenic fungi or diseased tissues. However, the amount of toxins is usually too low to perform further research such as structural identification. Moreover, many SMBGCs remain silent under standard fermentation conditions (Bergmann et al., 2007). Considering these limitations in toxin isolation and SMBGC identification, LaeA can be exploited as a tool to identify SMBGCs that might have an impact on virulence, allowing the correlation of specific SMBGCs with toxins and virulence (Perrin et al., 2007; Brakhage, 2013). Furthermore, genetic engineering, including overexpression of pathway-specific transcription factors (TFs), the use of strong inducible promoters, and manipulation of global regulators, could promote the identification of toxins produced by specific SMBGCs (Brakhage, 2013; Keller, 2019).

In this study, we showed that *VmLaeA* markedly affects virulence and toxic secondary metabolites (SMs) in *V. mali*. Transcriptomic and proteomic analyses found that *VmLaeA* regulates a large amount of transports and about half of the SMBGCs identified in *V. mali*. A non-ribosomal peptide synthesis (NRPS) gene cluster (*NRPS14*) was identified to be negatively regulated by *VmLaeA* and contribute to virulence. *VmLaeA* also seems to be involved in the regulation of pigment production by controlling specific polyketide synthesis (PKS) gene clusters. Our findings provide new insights on role of pathogenicity-related toxins in *V. mali*.

## Results

### Deletion of *VmLaeA* Markedly Attenuates Virulence of *Valsa mali*

**FIGURE 1:**
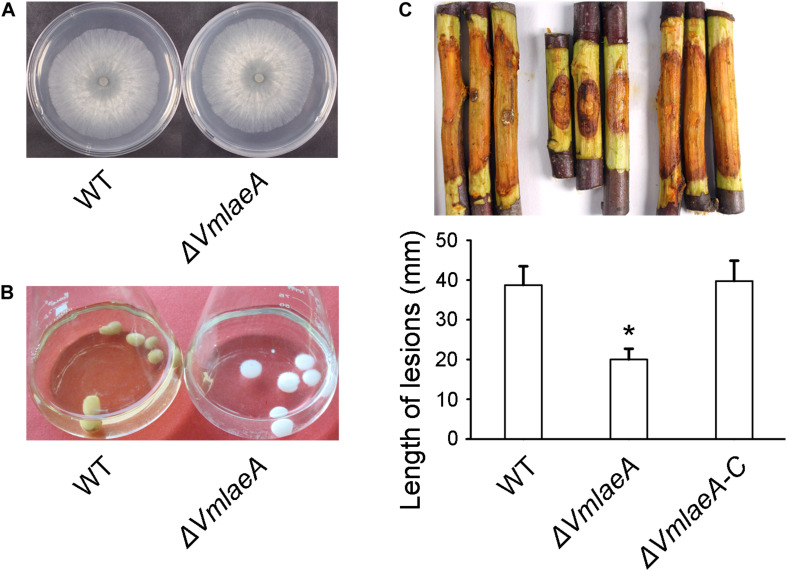
Growth and infection phenotype of *Valsa mali* WT and Δ*VmlaeA*. **(A)** Vegetative growth of *V. mali* WT and Δ*VmlaeA* mutant on potato dextrose agar (PDA) medium at 48 h. **(B)** Colony color of WT and Δ*VmlaeA* cultured in a synthetic liquid medium for 7 days. **(C)** Pathogenicity test of Δ*VmlaeA* on detached apple twigs. WT, Δ*VmlaeA*, and Δ*VmlaeA-C* were inoculated onto detached twigs of *Malus domestica* Borkh. cv. “Fuji” and incubated for 4 days. Bar graphs show the statistical analysis of at least three biological replicates, means ± SD are shown. Asterisk indicates a significant difference at *P* value <0.05.

LaeA was reported to influence fungal virulence and secondary metabolism in many filamentous fungi. *A. nidulans LaeA* homologous was identified in *V. mali* by BLAST and termed *VmLaeA*. To determine the potential roles of *VmLaeA*, we generated a deletion mutant of *VmLaeA* (Δ*VmlaeA*) and a complemented strain (Δ*VmlaeA-C*). Phenotypic analysis showed that Δ*VmlaeA* exhibited no apparent difference on vegetative growth after cultivation on potato dextrose agar (PDA) medium for 48 h (Figure 1A). However, under light conditions, Δ*VmlaeA* produced remarkably decreased amount of pycnidia compared with WT on PDA after 30 days. Under dark conditions, both strains produced very few pycnidia (Supplementary Figure 1). Intriguingly, the fermentation broth and mycelium pellets of WT turned yellow after 7 days of culture in a synthetic liquid medium, while those of Δ*VmlaeA* remained colorless (Figure 1B). These results suggest that the conidiation and pigmentation is affected by *VmLaeA*.

To investigate whether *VmLaeA* contributes to virulence in *V. mali*, WT, Δ*VmlaeA*, and Δ*VmlaeA-C* strains were inoculated onto detached apple twigs. As was shown, lesion length of Δ*VmlaeA* was reduced by approximately 48% after 4 days of cultivation in comparison with WT (Figure 1C). By contrast, the complemented strain Δ*VmlaeA-C* displayed a comparable lesion size as the WT (Figure 1C). These results indicate that *VmLaeA* acts as a crucial virulence factor.

### *VmLaeA* Affects *Valsa mali* Toxic SMs

**FIGURE 2:**
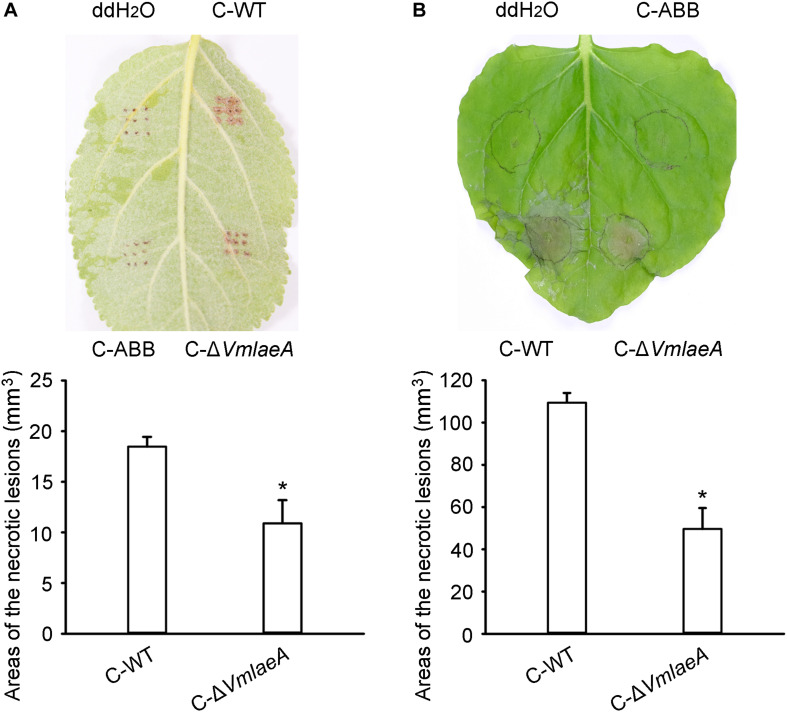
Toxicity of crude secondary metabolites (SMs) produced by *Valsa mali* WT and Δ*VmlaeA*. **(A)** Toxicity of crude SMs produced by WT and Δ*VmlaeA* on apple leaves. **(B)** Toxicity of crude SMs produced by WT and Δ*VmlaeA* on tobacco leaves. C-WT: crude SMs of WT; C-ABB: crude extracts of ABB medium; C-Δ*VmlaeA*: crude SMs of Δ*VmlaeA*. WT and Δ*VmlaeA* strains were grown in apple bark broth medium (ABB) for 10 days. Crude SMs of WT, Δ*VmlaeA*, and uninoculated medium (ABB) were obtained from culture filtrate by extraction with ethyl acetate and the dried solids were dissolved in ddH_2_O. Toxicity tests were carried out on apple leaves with a concentration of 50 mg/ml and on tobacco leaves with a concentration of 5 mg/ml. ddH_2_O and crude extracts of ABB were used as controls and pictures were taken at 24 h after treatment. Significant differences are indicated with asterisks (*P* value <0.05).

Considering the regulatory role of LaeA in toxin production in many phytopathogens, toxicity tests were conducted to investigate whether the production of SMs is affected by *VmLaeA*. We produced culture filtrates of WT, Δ*VmlaeA*, and uninoculated medium to obtain crude extracts of SMs. Toxicity of these crude SMs was tested on apple leaves as well as on tobacco leaves. Crude SMs of WT and Δ*VmlaeA* were able to induce necrotic symptom at 24 h after treatment. However, crude SMs from WT were more effective than those from Δ*VmlaeA* at the same concentration (Figure 2). On apple leaves, the areas of necrotic lesions caused by crude SMs of Δ*VmlaeA* amounted to just 59% of necrotic lesions caused by crude SMs of WT (Figure 2A). Similarly on tobacco leaves, necrotic lesions caused by crude SMs of Δ*VmlaeA* equated to only 45% of that cause by crude SMs of WT (Figure 2B). By contrast, the solvent and uninoculated medium control did not produce any noticeable lesions in the same time frame. These findings indicate that deletion of *VmLaeA* may lead to phyletic changes or reduction in toxic SMs.

### Transcriptomic and Proteomic Profiles of WT and Δ*VmlaeA*

**FIGURE 3:**
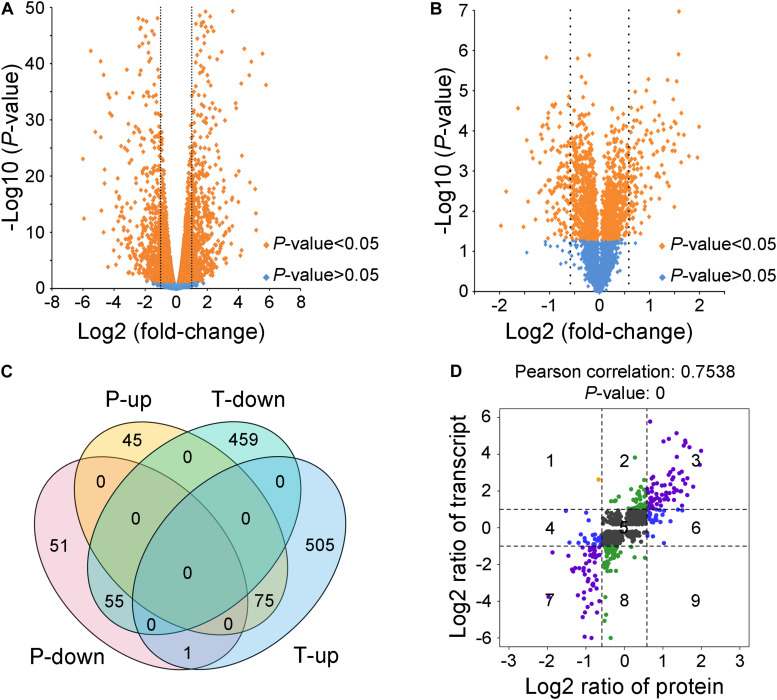
Differentially expressed genes (DEGs) and proteins (DEPs) in Δ*VmlaeA*. **(A)** Volcano plot of transcriptome data. The dotted lines represent fold change = 2/0.5, orange spots represent significant DEGs in Δ*VmlaeA*. **(B)** Volcano plot of proteome data. The dotted lines represent fold change = 1.5/0.67, orange spots represent significant DEPs in Δ*VmlaeA*. **(C)** Venn diagram of DEGs and DEPs in Δ*VmlaeA*. P-down: downregulated proteins in the proteome of Δ*VmlaeA*; P-up: upregulated proteins in the proteome of Δ*VmlaeA*; T-down: downregulated genes in the transcriptome of Δ*VmlaeA*; T-up: upregulated genes in the transcriptome of Δ*VmlaeA*. **(D)** Nine-quadrant diagram of DEGs and DEPs in Δ*VmlaeA*. The closer the Pearson correlation coefficient is to one, the higher the correlation between the two omics. *P* value <0.05 represents a significant correlation.

To explore how *VmLaeA* affects secondary metabolism and virulence in *V. mali*, RNA sequencing-based transcriptomic analysis and TMT-based proteomic analysis of both WT and Δ*VmlaeA* were performed. Totally, 1,095 of 9,945 genes identified from transcriptomes showed differential expression with fold changes >2 and *P* values <0.05 in Δ*VmlaeA*. Of these differentially expressed genes (DEGs), 514 were downregulated and 581 were upregulated (Figure 3A). Quantitative reverse transcription-PCR (qRT-PCR) analysis of randomly selected 10 downregulated DEGs and 10 upregulated DEGs displayed that these genes showed consistent up- or downregulation with their expression levels in transcriptomic analysis (Supplementary Figure 2). By comparing the proteomes of WT and Δ*VmlaeA*, 227 differentially expressed proteins (DEPs) of a total of 4,299 proteins identified (FDR < 0.01) were found, with fold changes >1.5 and *P* values <0.05. Among the 227 DEPs, 107 were decreased in abundance and 120 were increased in Δ*VmlaeA* (Figure 3B). The Venn diagram shows that transcriptomes and proteomes of WT and Δ*VmlaeA* share 131 DEGs (57.7% of all DEPs) in which 55 genes were downregulated and 75 genes were upregulated (Figure 3C). The Pearson correlation coefficient of the two omics is 0.7538 (*P* value = 0) which shows a significant positive correlation between the transcriptomic DEGs and proteomic DEPs (Figure 3D).

### *VmLaeA* Strongly Regulates Transport and Secondary Metabolism of *Valsa mali*

**FIGURE 4:**
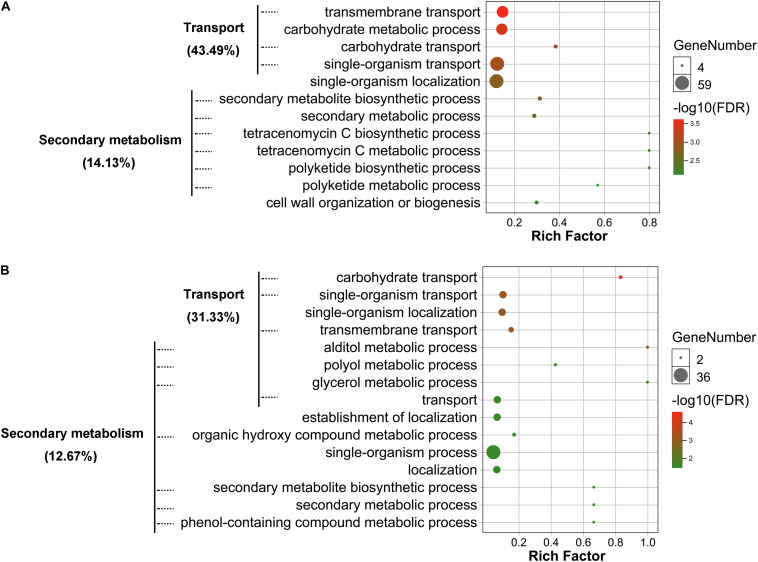
Gene ontology (GO) enrichment of biological process that differentially expressed genes (DEGs) and proteins (DEPs) were involved in. **(A)** Enriched biological process that transcriptomic DEGs were involved in. **(B)** Enriched biological process that proteomic DEPs were involved in. Processes with FDR <0.05 indicate that they were significantly enriched and are shown in the figures. Rich factor = DEGs (DEPs) number/total gene (protein) number identified from transcriptome or proteome of a certain process.

To determine potential functions of the DEGs and DEPs identified, annotation, functional classification, and enrichment analysis were performed. Gene ontology (GO) classification at level 2 GO terms was performed on transcriptomic DEGs and proteomic DEPs. The transcriptomic DEGs and proteomic DEPs showed very similar arrangement of GO classification (Supplementary Figure 3). The results of GO enrichment analysis showed that both transcriptomic DEGs and proteomic DEPs were mainly involved in transport and secondary metabolism-related biological processes (Figure 4 and Supplementary Tables 1 and 2). Transport-related biological processes that transcriptomic DEGs were involved in are consistent with the proteomic DEPs, with 43.49 and 31.33%, respectively, including transmembrane transport, carbohydrate transport, and single-organism transport (Figure 4). Secondary metabolism-related biological processes that transcriptomic DEGs were involved in include SM biosynthetic process, secondary metabolic process, polyketide biosynthetic process, tetracenomycin C metabolic process, and tetracenomycin C biosynthetic process, and the percentage of these DEGs is 14.13% (Figure 4A), whereas that of proteomic DEPs include alditol metabolic process, polyol metabolic process, glycerol metabolic process, organic hydroxy compound metabolic process, phenol-containing compound metabolic process, secondary metabolic process, and SM biosynthetic process, and the percentage of these DEPs is 12.67% (Figure 4B). The results of GO enrichment analysis demonstrate that *VmLaeA* conducts nearly uniform effects of transport and secondary metabolism in transcriptional and translational regulation.

In addition, a portion of CAZymes and proteases were regulated by *VmLaeA*. In total, 91 CAZyme genes were under the control of *VmLaeA*, including 40 upregulated and 51 downregulated (Supplementary Table 3). Also, 26 protease genes were regulated by *VmLaeA*, including 14 upregulated and 12 downregulated (Supplementary Table 4).

### Membrane Transporters Subject to Regulation of *VmLaeA*

**FIGURE 5:**
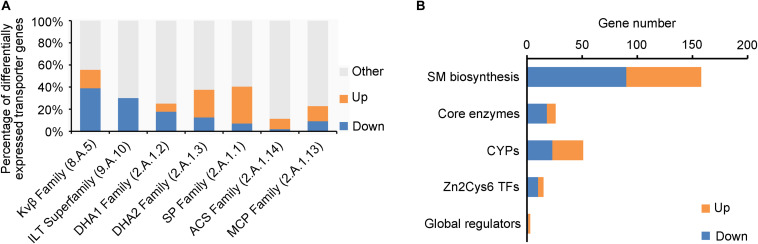
Enriched membrane transporter families and secondary metabolism-related genes from differentially expressed genes (DEGs) or proteins (DEPs). **(A)** Enriched membrane transporter families from DEGs or DEPs. Kvβ Family: the voltage-gated K^+^ channel β-subunit family; ILT Family: the iron/lead transporter family; DHA1 Family: the drug: H^+^ antiporter-1 (12 spanner) family; DHA2 Family: the drug: H^+^ antiporter-2 (14 spanner) family; SP Family: the sugar porter family; ACS Family: the anion: cation symporter family; MCP Family: the monocarboxylate porter family. **(B)** Enriched secondary metabolism-related genes from differentially expressed genes or proteins. CYPs: cytochrome p450s; Zn2Cys6 TFs: Zn2Cys6 transcription factors. Up: upregulated genes or proteins in Δ*VmlaeA* from transcriptomic and proteomic analyses; Down: downregulated genes or proteins in Δ*VmlaeA* from transcriptomic and proteomic analyses.

Fungal membrane transporters play an important role in counteracting the physiological impact of exogenetic antifungal compounds from host plant, other microbes, or synthesized by self-defense system and transport nutrients. Out of the 746 genomic membrane transporter genes of *V. mali*, 166 were differentially expressed in Δ*VmlaeA*, including 84 upregulated and 82 downregulated (Supplementary Table 5). The multidrug-resistant transporter (MFS superfamily) genes were markedly regulated by *VmLaeA*, including drug: H^+^ antiporter-1 family (DHA1 family), drug: H^+^ antiporter-2 family (DHA2 family), sugar porter family (SP family), anion: cation symporter family (ACS family), and monocarboxylate porter family (MCP family; Figure 5A). Especially, the DHA1 family is involved in multidrug resistance and infection. Of the 17 DHA1 family genes regulated by *VmLaeA*, 12 were under the positive regulation of *VmLaeA* and five of them (*VM1G_01696*, *VM1G_03590*, *VM1G_05084*, *VM1G_08059*, and *VM1G_08300*) had been proved significantly upregulated during infection. However, most genes of DHA2 family and SP family that were regulated by *VmLaeA* were under the negative regulation of *VmLaeA*, as 12 of 18 DHA2 family genes and 19 of 23 SP family genes were upregulated in Δ*VmlaeA*. The voltage-gated K^+^ channel β-subunit family (Kvβ family) that mediates signal transduction and iron/lead transporter family (ILT family) that is implicated in heavy metal binding all belong to ion channels. Of the 10 Kvβ family genes regulated by *VmLaeA*, seven were under the positive regulation of *VmLaeA*. Three ILT family genes accounted for 30% of *V. mali* ILT family and were under the negative regulation of *VmLaeA*. These results suggest that *VmLaeA* possibly affects virulence by specifically regulating multidrug-resistant transporters and signaling through specific ion channel transporters.

### About Half of the SMBGCs Are Regulated by *VmLaeA*

Genes involved in secondary metabolism consist of SM biosynthetic genes, core enzyme genes, cytochrome P450 (CYP) genes, Zn2Cys6 TF genes, and secondary metabolism-related global regulator genes (Figure 5B). Twenty-six core enzyme genes were found to be differentially expressed in Δ*VmlaeA*, including 8 upregulated and 18 downregulated (Figure 5B and Supplementary Table 6). Particularly, four of them that were positively regulated by *VmLaeA* were previously reported to be involved in infection, and one of them (*VM1G_04745*) has been proved to contribute to virulence. Fifty-one *CYPs* out of the 156 identified in *V. mali* were differentially expressed in Δ*VmlaeA*, including 28 upregulated and 23 downregulated (Figure 5B and Supplementary Table 7). Of them, five CYPs (three upregulated and two downregulated) belong to SMBGCs and one downregulated *CYP* gene (*VM1G_03094*) has been demonstrated previously to contribute to virulence. Furthermore, 15 genes for Zn2Cys6 TFs (5 upregulated and 10 downregulated) and three genes for global regulators were found to be differentially expressed in Δ*VmlaeA* (Figure 5B and Supplementary Tables 8 and 9). Of the 15 Zn2Cys6 TFs, five (two upregulated and three downregulated) belong to specific SMBGCs. In total, 158 SM biosynthetic genes (68 upregulated and 90 downregulated) were differentially expressed in Δ*VmlaeA*, including above 26 core enzyme genes, 5 CYP genes, 5 Zn2Cys6 TF genes, and 12 transporter genes. These results indicate that secondary metabolism-related genes are significantly regulated by *VmLaeA*.

To better understand the SM biosynthetic pathways regulated by *VmLaeA*, SMBGCs were re-predicted by antiSMASH (Medema et al., 2011) based on genome, transcriptome, and proteome data, and a total of 60 SMBGCs were identified in *V. mali* genome (Supplementary Table 10). All DEGs/DEPs involved in secondary metabolism were first matched with SMBGCs and clusters that contain two or more DEGs/DEPs were selected as differentially expressed SMBGCs (Supplementary Table 10). By analyzing differential expression of secondary metabolism-related genes in Δ*VmlaeA*, 31 differentially expressed SMBGCs were identified. Among these 31 SMBGCs, 17 gene clusters were downregulated, including 13 PKS gene clusters, 3 NRPS gene clusters, and 1 terpene synthesis gene cluster (Table 1). In addition, 14 upregulated gene clusters include seven PKS gene clusters, five NRPS gene clusters, and two NRPS/PKS hybrid gene clusters (Table 1). Especially, eight gene clusters showed completely consistent expression change at transcriptional and translational levels in Δ*VmlaeA*. Of the eight differentially expressed SMBGCs, five PKS gene clusters, including *PKS7*, *PKS11*, *PKS16*, *PKS23*, and *PKS31*, were downregulated, and *NRPS14*, *PKS39*, and hybrid gene cluster *NRPS/PKS33* were upregulated (Table 1). Over half of the SMBGCs are under the command of *VmLaeA*, and all these findings explain that *VmLaeA* is the crucial regulator of secondary metabolism in *V. mali*.

**TABLE 1:** Downregulated and upregulated secondary metabolite biosynthetic gene clusters (SMBGCs) in the Δ*VmlaeA* mutant.

**Type^a^**	**Downregulated**		**Upregulated**		**Description of the core enzyme**
	**Cluster name^b^**	**Core enzyme Gene ID**	**Cluster name^c^**	**Core enzyme Gene ID**	
PKS	*PKS5*	*VM1G_02156*	*PKS1*	*VM1G_00046*	Lovastatin diketide synthase
	*PKS9*	*VM1G_02824*	*PKS21*	*VM1G_04822*	
	*PKS20*	*VM1G_04801*	*PKS36*	*VM1G_08248*	
	*PKS34*	*VM1G_08019*	***PKS39***	*VM1G_09018*	
			*PKS45*	*VM1G_11041*	
	***PKS7***	*VM1G_02489*	*PKS13*	*VM1G_03186*	Polyketide synthase
	***PKS11***	*VM1G_03093*			
	*PKS15*	*VM1G_03589*			
	***PKS16***	*VM1G_03769*			
	***PKS23***	*VM1G_05383*			
	***PKS31***	*VM1G_07355*			
	*PKS17*	*VM1G_04331*	*PKS41*	*VM1G_09624*	Conidial yellow pigment biosynthesis polyketide synthase
	*PKS22*	*VM1G_04961*			
	*PKS49*	*VM1G_02661*			
NRPS	*NRPS4*	*VM1G_01528*			Linear gramicidin synthase
	*NRPS47*	*VM1G_11144*			Acyl-CoA synthetase family member 2, mitochondrial
	*NRPS10*	*VM1G_03054*	*NRPS27*	*VM1G_06774*	Non-ribosomal peptide synthetase
			*NRPS25*	*VM1G_06308*	Putative peroxisomal-coenzyme A synthetase
			*NRPS52*	*VM1G_08051*	Oxygen-dependent choline dehydrogenase
			***NRPS14***	*VM1G_03342*	Non-ribosomal peptide synthetase like
			*NRPS24*	*VM1G_05435*	
NRPS/PKS			*NRPS/PKS3*	*VM1G_01285*	Polyketide synthase-non-ribosomal peptide synthetase
			***NRPS/PKS33***	*VM1G_07481*	
TS	*TS60*	*VM1G_10824*			Dammaradiene synthase

*^*a*^PKS, polyketide synthesis; NRPS, non-ribosomal peptide synthesis; NRPS/PKS, hybrid non-ribosomal peptide and polyketide synthesis; TS, terpene synthesis.^*b*^Clusters marked in bold are downregulated in Δ*VmlaeA* at both transcriptional and translational level. Other clusters are downregulated only at the transcriptional level.^*c*^Clusters marked in bold are upregulated in Δ*VmlaeA* at both transcriptional and translational level. Other clusters are upregulated only at the transcriptional level.*

### *NRPS14* Contributes to *Valsa mali* Virulence

To further determine the functions of SMBGCs regulated by *VmLaeA*, all eight gene clusters regulated by *VmLaeA* at both transcriptional and translational levels were chosen for further functional validation experiments using gene knockout and overexpression analyses. We were able to generate deletion mutants of six of the eight gene clusters. These are Δ*Vmpks11*, Δ*Vmpks16*, Δ*Vmpks23*, Δ*Vmpks31*, Δ*Vmpks39*, and Δ*Vmnrps14*. Deletion mutants of *PKS7* and *NRPS/PKS33* could not be obtained. All deletion mutants were confirmed by Southern hybridization (Supplementary Figure 4A). Moreover, overexpression mutants of the six gene clusters (Δ*Vmpks11OE*, Δ*Vmpks16OE*, Δ*Vmpks23OE*, Δ*Vmpks31OE*, Δ*Vmpks39OE*, Δ*Vmnrps14OE*-6, and Δ*Vmnrps14OE*-13) were conducted through overexpressing the TF gene of corresponding clusters. Δ*Vmnrps14OE*-6 and Δ*Vmnrps14OE*-13 are the two overexpression mutants of cluster *NRPS14*. Overexpression mutants were verified by qRT-PCR and generally upregulated about threefolds (Supplementary Figure 4B).

**FIGURE 6:**
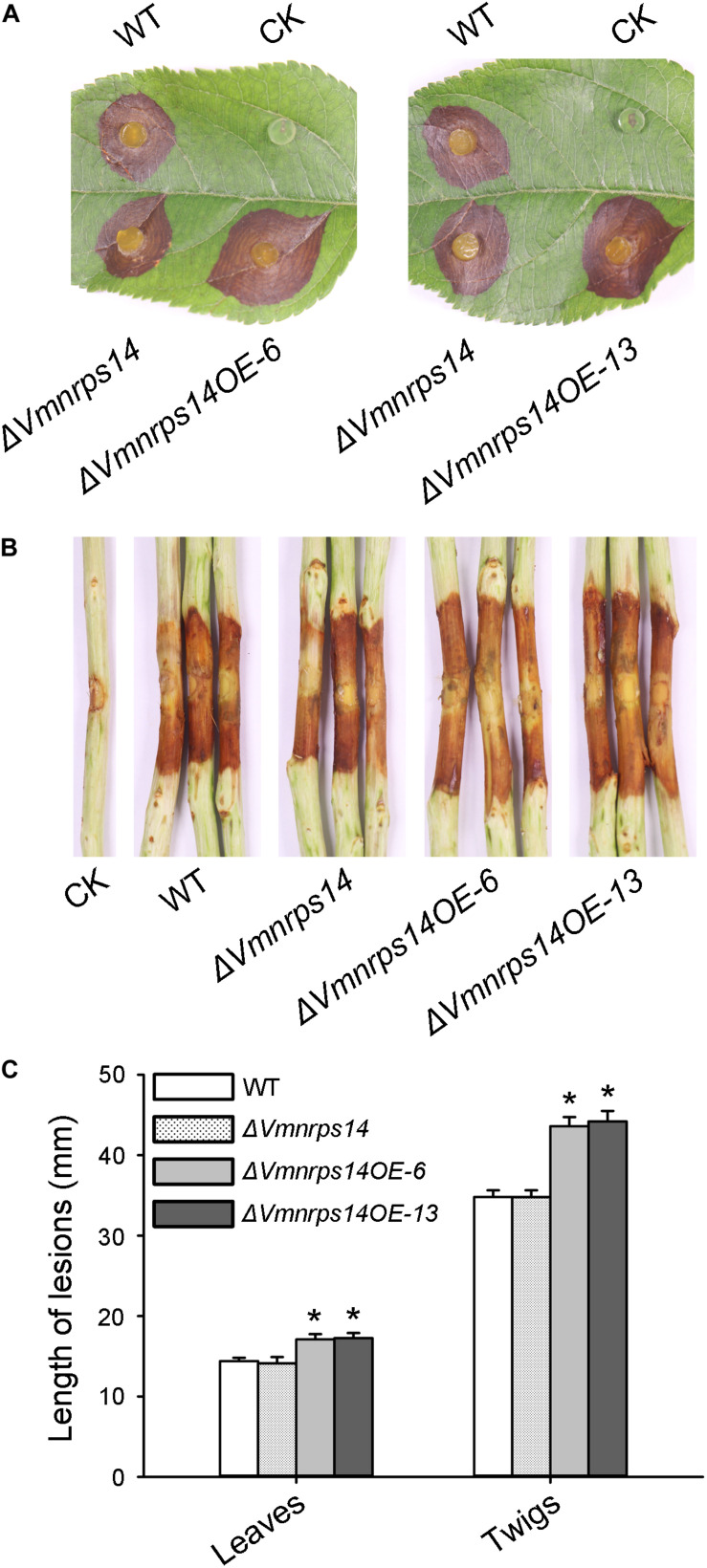
Infection assays of WT, Δ*VmNRPS14*, and Δ*Vmnrps14-OE*. **(A)** Pathogenicity test of Δ*VmNRPS14* and Δ*Vmnrps14-OE* on detached apple leaves. WT, Δ*VmNRPS14*, and Δ*Vmnrps14-OE* were inoculated on detached leaves of *Malus domestica* Borkh. cv. “Fuji” and were cultured for 3 days. **(B)** Pathogenicity test of Δ*VmNRPS14* and Δ*Vmnrps14-OE* on detached apple twigs. WT, Δ*VmNRPS14*, and Δ*Vmnrps14-OE* were inoculated on detached twigs of *Malus domestica* Borkh. cv. “Fuji” and were cultured for 4 days. **(C)** Bar graph of lesions length cause by WT, Δ*VmNRPS14*, and Δ*Vmnrps14-OE*. In bar graphs, means ± SD of at least three biological replicates are shown. Significant differences are indicated with asterisks (*P* value <0.05).

**FIGURE 7:**
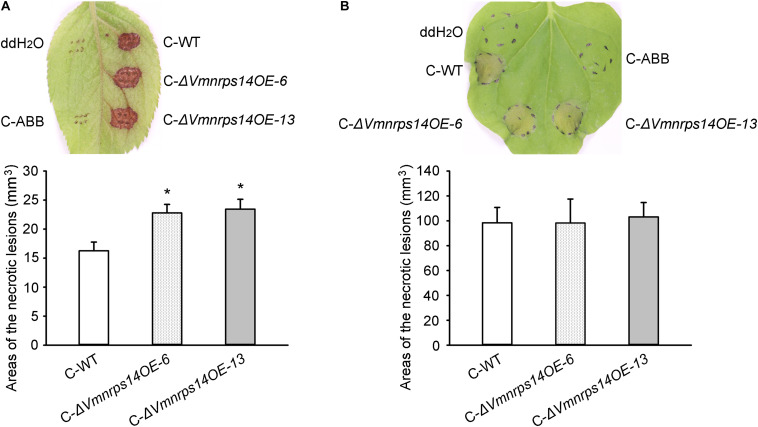
Toxicity of crude secondary metabolites (SMs) produced by *Valsa mali* WT and the overexpression mutants of *NRPS14*. **(A)** Toxicity of crude SMs produced by WT and the overexpression mutants of *NRPS14* on apple leaves. **(B)** Toxicity of crude SMs produced by WT and the overexpression mutants of *NRPS14* on tobacco leaves. C-WT: crude SMs of WT; C-ABB: crude extracts of ABB medium; C-Δ*Vmnrps14OE-6*: crude SMs of Δ*Vmnrps14OE-6*; C-Δ*Vmnrps14OE-13*: crude SMs of Δ*Vmnrps14OE-13*. WT and the overexpression mutants of *NRPS14* (Δ*Vmnrps14OE-6* and Δ*Vmnrps14OE-13*) were grown in apple bark broth medium (ABB) for 10 days. Crude SMs were obtained from culture filtrate by extraction with ethyl acetate and the dried solids were dissolving in ddH_2_O. Toxicity tests were carried out on apple leaves with a concentration of 50 mg/ml and on tobacco leaves with a concentration of 5 mg/ml. ddH_2_O and crude extracts of ABB were used as controls and pictures were taken at 24 h after treatment. Significant differences are indicated with asterisks (*P* value <0.05).

To evaluate the roles of the six gene clusters on vegetative growth, all deletion and overexpression mutants along with the WT were cultured on PDA for 48 h to measure mycelia growth. All deletion and overexpression mutants showed no significant difference on vegetative growth (Supplementary Figure 5). However, Δ*Vmpks11OE*, Δ*Vmpks16OE*, Δ*Vmpks23OE*, and Δ*Vmpks31OE* exhibited obvious accumulation of pigments after cultivation on PDA medium for 5 days (Supplementary Figure 6). To determine whether *PKS11*, *NRPS14*, *PKS16*, *PKS23*, *PKS31*, or *PKS39* are involved in pathogenesis, we next determined the virulence phenotype of their mutants. The results showed that all deletion mutants of the six gene clusters had no significant influence on *V. mali* virulence (Figure 6 and Supplementary Figure 7). Consistently, overexpression of *PKS11*, *PKS16*, *PKS23*, *PKS31*, and *PKS39* also showed no significant influence on fungal virulence (Supplementary Figure 7). However, when *NRPS14* was overexpressed, the lesion diameters were increased about 19.35% on detached apple leaves and 26.15% on detached apple twigs (Figure 6). To investigate whether the overexpression of *NRPS14* affects the toxicity of SMs produced by *V. mali*, the crude SMs of WT and two overexpression mutants of *NRPS14* (Δ*Vmnrps14OE-6* and Δ*Vmnrps14OE-13*) were produced to conduct toxicity tests. The results showed that toxicity of the crude SMs of Δ*Vmnrps14OE-6* and Δ*Vmnrps14OE-13* increased 40 and 44%, respectively, in comparison with that of WT on apple leaves (Figure 7A). However, on tobacco leaves, the necrotic lesions caused by the crude SMs of WT and two overexpression mutants of *NRPS14* are of uniform size at the same concentration (Figure 7B). The *NRPS14* cluster contains 20 genes predicted by antiSMASH and six of these genes are significantly upregulated in Δ*VmlaeA*. Especially, *VM1G_03342*, the core enzyme gene of *NRPS14*, is upregulated by 8.17-fold at the transcriptional level in Δ*VmlaeA* (Supplementary Table 10). These results indicate that *NRPS14* is negatively regulated by *VmLaeA* and contributes to virulence. Toxicity tests showed that overexpression of *NRPS14* affected the production of toxic SMs of *V. mali.*

## Discussion

Toxins produced by plant pathogenic fungi are often crucial determinants of plant disease, as toxins can reproduce at least partial disease symptoms and non-toxic mutants are non-pathogenic or show reduced virulence (Richard, 2007). Previous research on the *V. mali* genome and transcriptome suggested that biosynthetic pathways of toxin are probably involved in virulence. Therefore, exploration of *V. mali* SMBGCs would facilitate revealing the pathogenesis mechanisms of *V. mali*. In this study, we investigated the function of *VmLaeA* and profiled transcriptomes and proteomes of *V. mali* WT and Δ*VmlaeA.* Absence of *VmLaeA* caused markedly compromised virulence of *V. mali*. Analysis of the two omics showed *VmLaeA* powerfully regulates secondary metabolism-related genes and SMBGCs.

LaeA has a minor impact on fungal vegetative growth but participates in light-dependent development through the interaction with the regulator of sporogenesis VosA (Bayram et al., 2008). Despite light triggering asexual development, deficiency of *VmLaeA* cut off the interaction between LaeA and VosA, corresponding to the decrease of pycnidia in Δ*VmlaeA* whether in dark or light. Deletion of *laeA* caused a loss of mycelial pigmentation in *Aspergillus* spp. as it was first identified as a global regulator (Bok and Keller, 2004). Similarly, deletion of *LaeA* in *Pestalotiopsis microspore* led to the retardation of pigmentation (Akhberdi et al., 2018). Consistent with these findings, the fermentation broth and mycelium pellets of Δ*VmlaeA* showed reduced pigmentation compared with WT in a synthetic liquid medium. However, *LaeA* is not always affecting pigmentation. In *Aspergillus fumigatus*, deletion of *laeA* did not affect pigmentation in strains B-5233 and AF293 (Sugui et al., 2007). More notably, overexpression of four PKS clusters (*PKS11*, *PKS16*, *PKS23*, and *PKS31*) that were positively regulated by *VmLaeA* led to obvious accumulation of pigments and the core enzymes of these four clusters were all identified as conidial yellow pigment biosynthesis polyketide synthases. Conidial pigmentation provides protection against desiccation, ultraviolet light, ionizing radiation, and oxidative stress and contributes to survival of fungi (Belozerskaya et al., 2017; Chang et al., 2020). Controlling conidial pigment biosynthesis could be a way that *VmLaeA* help *V. mali* cope with harsh environments.

With the identification of *LaeA* in plenty of pathogenic fungi, *LaeA* was regarded as a general virulence factor. Deletion of *LaeA* weakened the infection ability of *A. ochraceus* on pear (Wang et al., 2019). In maize pathogen *C. heterostrophus*, both T-toxin–mediated super virulence and basic pathogenic ability were affected when *ChLae1* was deleted or overexpressed (Wu et al., 2012). *LaeA* mutants of two *P. expansum* isolates (Pe-21 from Israel and Pe-T01 from China) showed differential reduction in disease severity (Kumar et al., 2017). Deletion of *BcLAE1* in *B. cinerea* resulted in the mutant losing the ability to colonize the host tissue and to produce oxalic acid (Schumacher et al., 2015). Similar in *V. mali*, deletion of *VmLaeA* led to greatly reduced virulence on apple twigs. As a conserved and crucial virulence factor, further exploration of the regulatory mechanism by *VmLaeA* is of great importance to reveal the pathogenic mechanism of *V. mali*.

Transcriptomic and proteomic analyses indicated plenty of genes that are controlled by *VmLaeA* seem to be involved in infection-related processes, including membrane transporter genes, CAZyme genes, and protease genes. Membrane transporters could counteract the physiological impact of host antifungal defense compounds and transport nutrients. Our research found DHA1 transporters, a member of MFS superfamily, were positively regulated by *VmLaeA*. DHA1 transporters were significantly upregulated during *V. mali* infection and involved in multidrug resistance (Meredith and Christian, 2008; Sá-Correia et al., 2009; Yin et al., 2015). In addition, 91 CAZyme genes and 26 protease genes were found to be under the control of *VmLaeA*. It was reported that CAZymes, in particular cell wall–degrading enzymes (CWDEs) and some proteases, target structural materials of plant cell wall and degrade cell walls to promote infection and colonization (Dow et al., 1998; Dunaevsky et al., 2007; Mohnen, 2008). These results indicate *VmLaeA* probably regulates virulence by controlling multiple infection-related processes including counteracting host antifungal compounds, cell wall degradation, killing host cells, and nutrient uptake from host cell and transport.

Toxicity and pathogenicity tests indicate that deletion of *VmLaeA* leads to reduced toxic SMs and decreased virulence in *V. mali*. In phytopathogenic fungi, LaeA modulates virulence by regulating the expression of many toxin biosynthetic gene clusters and the production of corresponding toxins. In *P. expansum*, *LaeA* affects patulin production and patulin-modulated virulence by positively regulating the expression of the patulin biosynthetic gene cluster (Kumar et al., 2017). Analysis of *V. mali* secondary metabolism-related genes revealed that more than half of the SMBGCs are under the control of *VmLaeA*. Of these SMBGCs, *NRPS14*, negatively regulated by *VmLaeA*, was shown to affect virulence. It was reported that many specialized SMBGCs keep silent and are activated only under some conditions by specific induction (Rutledge and Challis, 2015). For instance, the silent trichosetin gene cluster in *F. fujikuroi* is activated by the overexpression of the cluster-specific TF gene TF22 and then produces trichosetin (Janevska et al., 2017). This may explain why cluster *NRPS14* influences virulence only when it is overexpressed but not deleted. The phenomenon that crude SMs of Δ*Vmnrps14OE-6* and Δ*Vmnrps14OE-13* exhibited increased toxicity on apple leaves instead on tobacco leaves suggested that overexpression of *NRPS14* may lead to phyletic change or counts increase in toxic SMs, and the changed SMs could be host specific, like T-toxin in *C. heterostrophus* (Wu et al., 2012). Although the overexpression of *NRPS14* was only verified by traditional PCR and RT-qPCR, current results including virulence and toxicity tests also proved that the gene was overexpressed. It seems there is still a contradiction as deletion of *VmLaeA* increased the expression of virulence-related *NRPS14* but showed decreased virulence. As a matter of fact, reduction in virulence of Δ*VmlaeA* is most likely due to the combined effects of multiple virulence factors regulated by *VmLaeA.* For instance, one NRPS gene *VM1G_04745* that was positively regulated by *VmLaeA* had been shown to contribute to virulence (Ma et al., 2016). *VmLaeA* also positively regulated two genes (*VM1G_01526* and *VM1G_01527*) that belong to a virulence-related gene cluster *NRPS4* (data not shown). CYPs can catalyze the conversion of hydrophobic intermediates of secondary metabolic pathways. One CYP gene *VM1G_03094* that was positively regulated by *VmLaeA* had been proved to contribute to virulence (Gao et al., 2016). In addition, reduction in toxicity of the crude SMs of Δ*VmlaeA* also implied other virulence-related SMBGCs were involved. These SMBGCs might contribute to virulence by producing toxins. However, further research of toxins produced by these SMBGCs is still needed.

The phenomenon that deletion or silencing of some specific SMBGCs or regulatory genes may improve the biosynthesis of other SMs is known as cross-regulation (cross-talk) of SM biosynthetic pathways (Bergmann et al., 2010; Brakhage, 2013; Liu et al., 2013). *P. chrysogenum* mutants lacking the penicillin gene cluster produce increasing amounts of PR-toxin, and mutants of *P. roqueforti* silenced in the PR-toxin genes produce large amounts of mycophenolic acid (Martín, 2017). The LaeA-velvet complex could mediate pathway cross-talk of secondary metabolism. In Δ*VmlaeA*, 17 SMBGCs were downregulated and 14 were upregulated. Cross-talk may implicate in regulation of *V. mali* secondary metabolism and increased the complicacy of SMBGC expression. Apart from toxins and pigments, some SMBGCs may also be involved in the synthesis of SMs which are responsible for signal transduction, such as kojic acid that acts as a macrophage activator in *Aspergillus* spp. (Dufour and Rao, 2011; Rodrigues et al., 2011). These may be part of the reasons why some SMBGCs regulated by LaeA showed no effect on virulence after deletion and overexpression in our study.

In summary, *VmLaeA* is a crucial virulence factor of *V. mali*. SMBGCs are largely regulated by *VmLaeA* and contribute to virulence. SMBGCs that are involved in virulence provide genetic resources for elucidating pathogenicity-related toxins in *V. mali*.

## Materials and Methods

### Stains, Media, and Culture Conditions

The WT strain of *V. mali* was obtained from the stock culture of the Laboratory of Pathogen Biology and Integrated Control of Fruit Tree Diseases, College of Plant Protection, Northwest A&F University, Yangling, Shaanxi, China. Strains used in this study were cultured on PDA medium (20% potato extract, 2% glucose, and 1.5% agar) at 25°C. Potato dextrose broth medium (PDB; 20% potato extract, 2% dextrose) was used for cultivating mycelium used for RNA-sequencing and proteomic analysis. A synthetic liquid medium (10 g of sucrose, 2 g of L-asparagine monohydrate, 1 g of potassium dihydrogen phosphate, 0.5 g of magnesium sulfate heptahydrate, 0.88 g of zinc sulfate heptahydrate, 1.5 mg of ferric nitrate monohydrate, 0.44 mg of manganese sulfate pentahydrate, 5 μg of biotin, 100 μg of thiamine in 1 L) was used for fermentation to observe the color change of the mycelium. Apple bark broth (ABB) medium (30% apple branch extract, 0.2% yeast extract powder, 0.5% sucrose) was used for fermentation to extract crude SMs.

### RNA-Sequencing Analysis of WT and Δ*VmlaeA*

#### RNA Preparation

Three biological replicates of WT and Δ*VmlaeA* were cultivated in PDB at 25°C and 100 rpm for 48 h. Mycelium was collected and frozen in liquid nitrogen. Total RNA was extracted using RNAiso Plus (Takara, Shiga, Japan) according to the manufacturer’s protocol. RNA integrity number (RIN) of all RNA samples was assessed using a Bioanalyzer (Agilent Technologies, Santa Clara, CA, United States) and samples of which the RIN are greater than six were considered to be of sufficient quality.

#### RNA-Sequencing

The cDNA libraries were prepared using the TruSeq Stranded mRNA Library Preparation kit (Illumina, San Diego, CA, United States). The cDNA was sequenced on a HiSeq2000 platform (Illumina). After filtering out low-quality reads (Phred ≤ 20) and adaptors, clean reads were mapped against predicted transcripts of the *V. mali* genome using Tophat v2.0.4 (Trapnell et al., 2009).

### TMT-Based Proteomics Analysis of WT and Δ*VmlaeA*

#### Protein Extraction

Extraction and purification of protein from mycelium was carried out according to the trichloroacetic acid (TCA)/acetone-sodium dodecyl sulfate (SDS)/phenol extraction method (Wang et al., 2003) with little modification. Mycelium dried at low temperature was ground into powder in liquid nitrogen. The ground powder was suspended in acetone containing 10% TCA and 0.2% dithiothreitol and allowed to stand for 2 h. After purification with methanol, the precipitate was extracted using SDS/phenol extraction buffer for 5 min on ice. The upper phase was transferred and precipitated by acetone containing 100 mmol ammonium acetate per liter. Proteins were obtained by centrifugation. SDS-PAGE was employed to control the quality of the extracted proteins.

#### Protein Digestion, TMT Labeling, and NanoLC-MS/MS Analysis

Protein samples were digested using sequencing-grade trypsin for 24 h at 37°C. Trypsin-digested peptides were labeled with TMT 10-plex reagents, using 126-tag, 127N-tag, 127C-tag for the three biological replicates of the WT, and 129C-tag, 130N-tag, 130C-tag for the three biological replicates of Δ*VmlaeA*, respectively. The tag-labeled samples were then mixed followed by the first dimensional high pH reverse-phase (hpRP) separation of tryptic peptide mixtures using an UltiMate 3000 MDLC platform (Thermo-Dionex, Sunnyvale, CA, United States). A total of 12 fractions were collected and dried for further nanoLC-MS/MS analysis. The nanoLC-MS/MS analysis was performed by an Orbitrap Fusion (Thermo-Fisher Scientific, San Jose, CA, United States) mass spectrometer equipped with nano ion source using high-energy collision dissociation coupled with auto-sampler injection and NanoLC RP (NanoLC, 75 μm ID column). A 120-min NanoLC gradient on one LC fraction was used for MS/MS analysis as described previously (Qin et al., 2019). All MS and MS/MS raw spectra were processed and searched using Sequest HT software within the Proteome Discoverer 1.4 (PD1.4; Thermo). The *V. mali* protein sequence database containing 11,218 entries downloaded on NCBI^1^ were used for database searches. The default search settings used for 10-plex TMT quantitative processing and protein identification in PD1.4 searching software were two mis-cleavages for full trypsin with fixed carbamidomethyl modification of cysteine, fixed 10-plex TMT modifications on lysine and N-terminal amines, and variable modifications of methionine oxidation and deamidation on asparagines/glutamine residues. The peptide mass tolerance and fragment mass tolerance values were 10 ppm and 50 mDa, respectively. Identified peptides were filtered for maximum 1% FDR using the Percolator algorithm in PD 1.4 along with additional peptide confidence set to high. The TMT10plex quantification method within Proteome Discoverer 1.4 software was used to calculate the reporter ratios with mass tolerance ±10 ppm without applying the isotopic correction factors. Only peptide spectra containing all reporter ions were designated as “quantifiable spectra” and used for peptide/protein quantitation. A protein ratio was expressed as a median value of the ratios for all quantifiable spectra of the unique peptides pertaining to that protein. A precursor co-isolation filter of 50% was also applied for minimizing ratio compression caused by co-isolation of precursor ions. For each relative ratio group, normalization on protein median was applied. The comparison between groups was undertaken with Microsoft Excel software.

### Analysis of Transcriptome and Proteome Data

Transcriptomic DEGs of Δ*VmlaeA* were analyzed using the DESeq package (Anders and Huber, 2010) with fold change >2 (beta value >1 or <−1) and FDR ≤ 0.05 as thresholds. The database search of proteomic data was performed by Proteome Discoverer 2.1 (PD 2.1) with the Sequest HT search engine for both protein identification and quantification against the protein database of *V. mali* which consists of 11,218 sequence entries. Peptide identification was filtered by FDR less than 1% and at least two unique peptides were required. Differentially expressed proteins were determined by a 1.5-fold change cut-off. Blast2go (Bioinformatics Department, Valencia, Spain) and OmicShare^2^ were used for the GO and KEGG annotations and functional classification of identified genes. SMBGCs were predicted by antiSMASH.

### Gene Deletion and Overexpression

#### Gene Deletion and Complementation

*VmLaeA* and the core enzyme genes of cluster *PKS7*, *PKS11*, *NRPS14*, *PKS16*, *PKS23*, *PKS31*, *NRPS/PKS33*, and PKS*39* were selected to construct deletion mutants. The gene knockout cassettes were assembled by double-joint PCR with upstream and downstream flanking sequences of the target gene and *neo* gene as selective marker (Yu et al., 2004; Supplementary Figure 8). The gene knockout cassettes were then transformed into protoplasts of *V. mali* mediated by polyethylene glycol (Gao et al., 2011). Putative deletion mutants filtered by selection with geneticin were confirmed by PCR and further confirmed by Southern blot hybridization using the DIG DNA Labeling and Detection Kit II (Roche, Mannheim, Germany). Gene complementation was conducted by cloning the target genes into plasmid PDL2 by the yeast gap repair approach and then transforming the recombined plasmids into the respective target gene deletion mutant (Bruno et al., 2004; Zhou et al., 2012). The complemented mutants were finally confirmed by PCR. All primers used in gene deletion and complementation are listed in Supplementary Table 11.

#### Overexpression of the TF Genes

The TF genes of cluster *PKS11*, *NRPS14*, *PKS16*, *PKS23*, *PKS31*, and *PKS39* were predicted by antiSMASH (Medema et al., 2011) and cloned into plasmid PDL2. The recombinant plasmids were then transformed into protoplasts of *V. mali* after sequencing. Transformants were firstly filtered by selection with hygromycin B and PCR was conducted to select overexpression mutants for each TF gene. Afterward, RNA of WT and the overexpression mutants were extracted and used for qRT-PCR analysis to determine the overexpression of the TF genes. *G6PDH* was used as reference gene (Yin et al., 2013) and relative expression levels were calculated with the 2^–ΔΔCT^ method (Livak and Schmittgen, 2001).

### Phenotypic Analysis and Pathogenicity Assays

WT, deletion, complemented, and overexpression mutants were inoculated onto PDA plates and the growth, color, and conidia formation were observed at 48 h, 5, and 30 days. Pathogenicity assays were conducted by inoculating WT and mutants on detached leaves or twigs of *Malus domestica* Borkh. cv. “Fuji” and cultivation for 3 days on leaves or 4 days on twigs at 25°C followed by lesion measuring and data analysis (Wei et al., 2010). Each experiment was replicated at least three times.

### Extraction of Crude Extracts From Culture Filtrate and Toxicity Tests

WT and Δ*VmlaeA* were cultivated in ABB medium at 25°C and 100 rpm for 10 days. Culture filtrates of WT, Δ*VmlaeA*, and ABB medium were dried at low temperature and extracted with ethyl acetate three times. The organic phase was concentrated to obtain crude extracts of SMs. All crude extracts were dissolved to 50 mg/ml in ddH_2_O and tested on apple leaves with the leaf puncture method and tested on tobacco leaves by infiltration with the concentration of 5 mg/ml (Sugawara et al., 1987; Wang C. et al., 2014). ABB crude extract–treated and ddH_2_O-treated samples were kept as control and symptoms were carefully examined 48 h every 12 h. The pictures were taken at 24 h after treatment with a ruler included. ImageJ software was used to calculate the areas of necrotic lesions. At least three biological repeats of necrotic lesions were used in the statistical analysis.

## Data Availability Statement

The transcriptomic data has been uploaded to SRA database (accession: PRJNA666017). The proteomics data have been deposited to the ProteomeXchange Consortium via the PRIDE partner repository with the dataset identifier PXD021783.

## Author Contributions

YF, YW, and HD performed the experiment. YF, ZY, and LX analyzed the results. YF, ZY, YW, LX, NW, and LH composed the article. All authors contributed to the article and approved the submitted version.

## Conflict of Interest

The authors declare that the research was conducted in the absence of any commercial or financial relationships that could be construed as a potential conflict of interest.

## Footnotes

1 https://www.ncbi.nlm.nih.gov/

2 https://www.omicshare.com/tools/
