# Supplementary material for: LaeA Controls Virulence and Secondary Metabolism in Apple Canker Pathogen Valsa mali
Source: Front Microbiol. 2020 Nov 5;11:581203. doi: 10.3389/fmicb.2020.581203 (PMC7674932; doi:10.3389/fmicb.2020.581203)
Supplement: Supplementary file 2 [file Data_Sheet_2.docx]

**
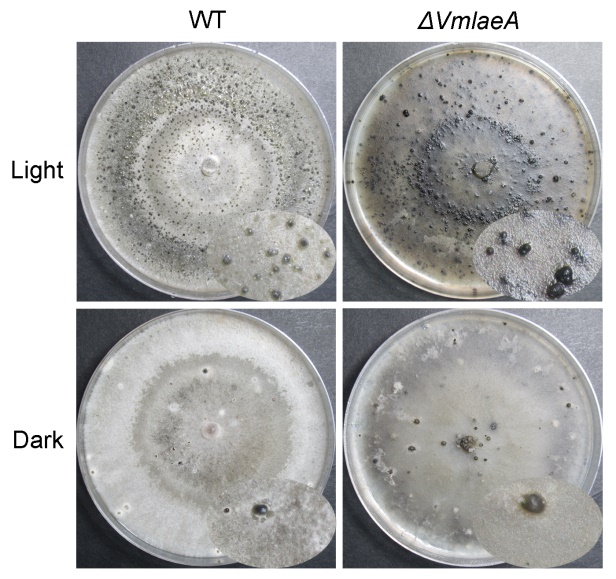
**

**Supplementary Figure 1. Conidiation of** ***Valsa mali* WT and *ΔVmlaeA* under light and dark conditions.** WT and *ΔVmlaeA* strain were cultivated on PDA medium for 30 days under light and dark conditions, respectively.


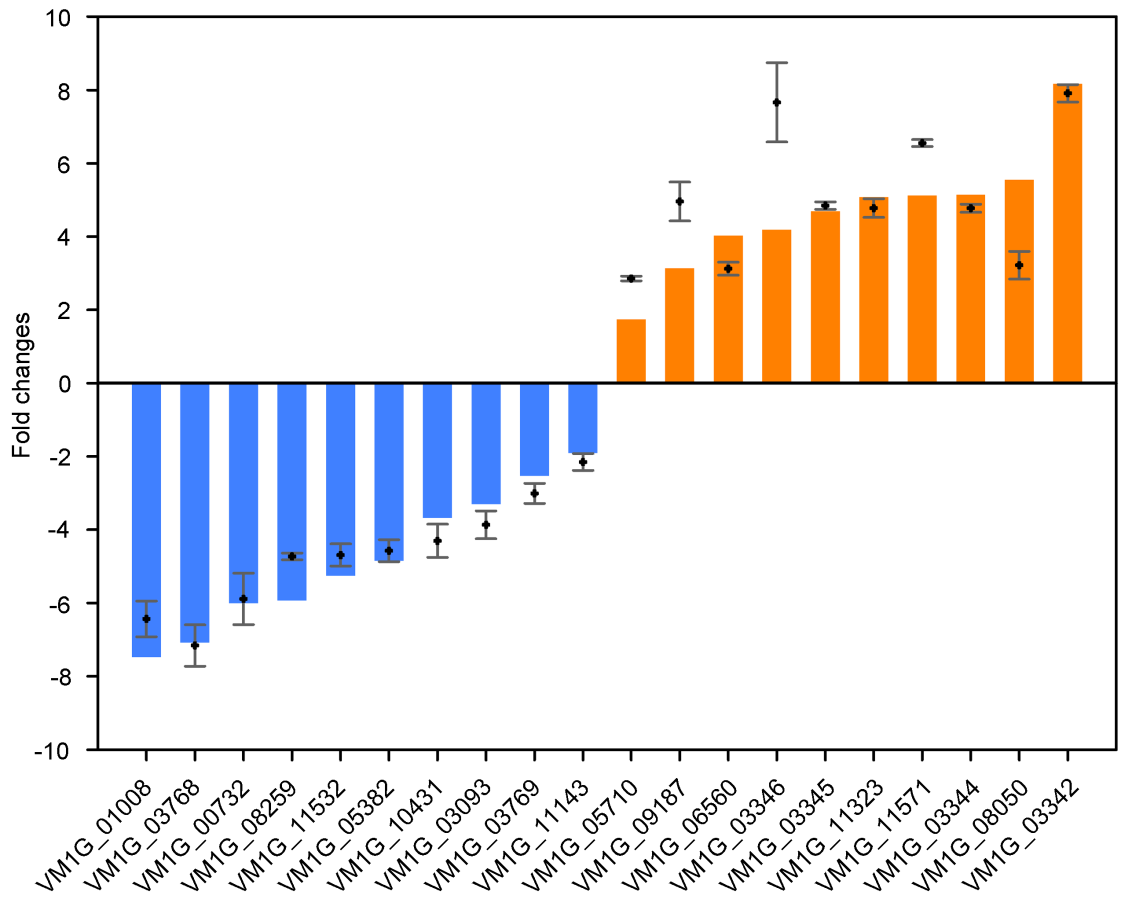


**Supplementary Figure 2. Verification of 20 differentially expressed genes (DEGs) from transcriptomic analysis by RT-qPCR.** The bars showed the fold changes of the 20 DEGs from transcriptomic analysis. Scatters with error bars presented the fold changes of the 20 DEGs verified by RT-qPCR. Minus fold change represent the gene was down-regulated in *ΔVmlaeA* for corresponding multiple. The RT-qPCR experiments were repeated three times.


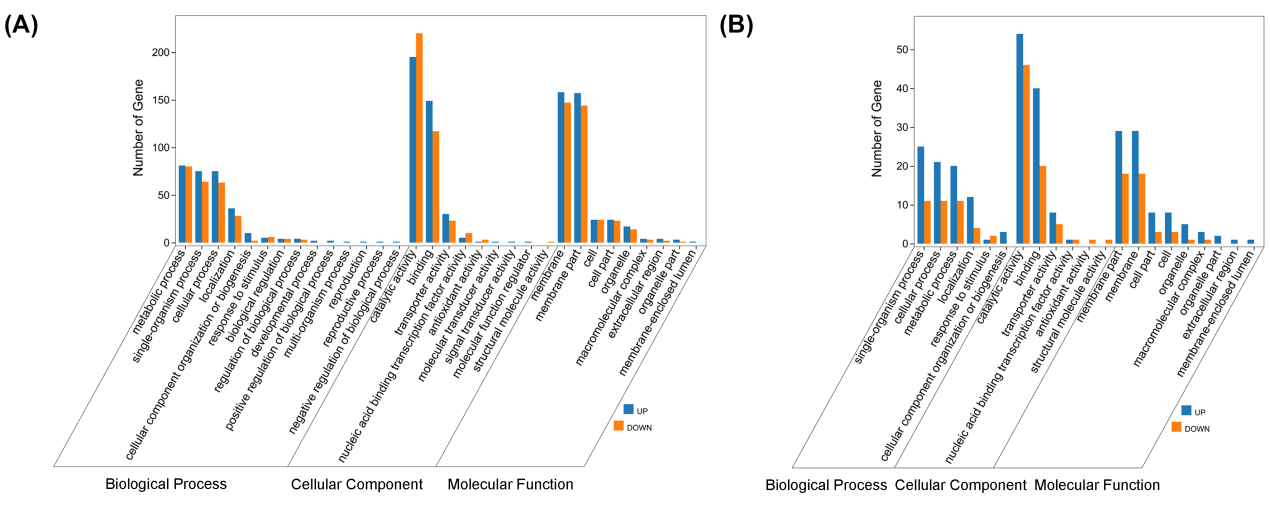


**Supplementary Figure 3. Gene ontology (GO) classification of differentially expressed genes (DEGs) and proteins (DEPs) at level 2 GO terms.** **(A)** GO classification of transcriptomic DEGs at level 2 GO terms. **(B)** GO classification of proteomic DEPs at level 2 GO terms.


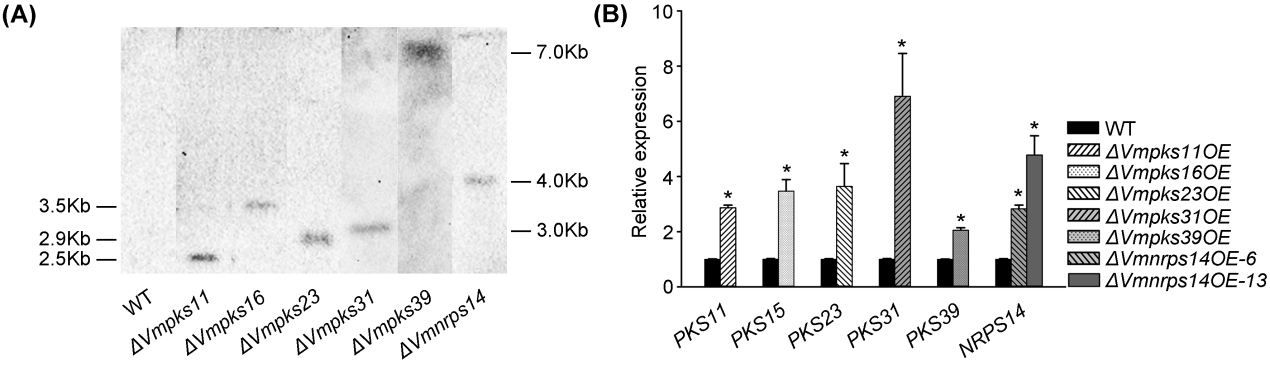


**Supplementary Figure 4. Confirmation of deletion and overexpression mutants of cluster *PKS11*, *NRPS14*, *PKS16*, *PKS23*, *PKS31* and *PKS39.* (A)** Southern blots of genomic DNA of WT and the core enzyme gene deletion mutants of cluster *PKS11*, *NRPS14*, *PKS16*, *PKS23*, *PKS31* and *PKS39* hybridized with *neo* gene probe. **(B)** Relative expression levels of the transcription factor gene of cluster *PKS11*, *NRPS14*, *PKS16*, *PKS23*, *PKS31* and *PKS39* in WT and their overexpression mutants. Asterisks represent significant difference in expression levels (*P*-value < 0.05).

**
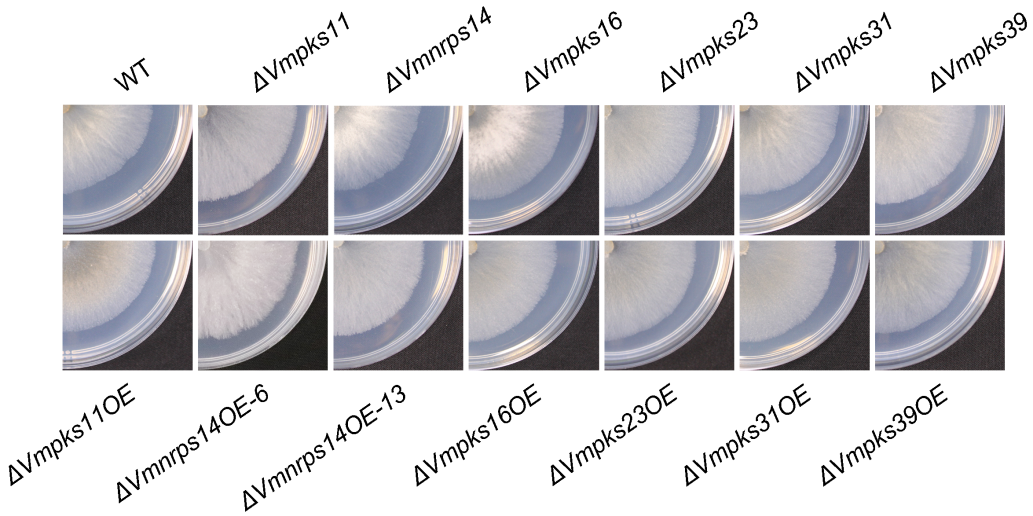
**

**Supplementary** **Figure 5. Vegetative growth of WT, deletion and overexpression mutants of cluster *PKS11*, *NRPS14*, *PKS16*, *PKS23*, *PKS31* and *PKS39*.** All stains were inoculated on PDA and cultured for 48 h.

**
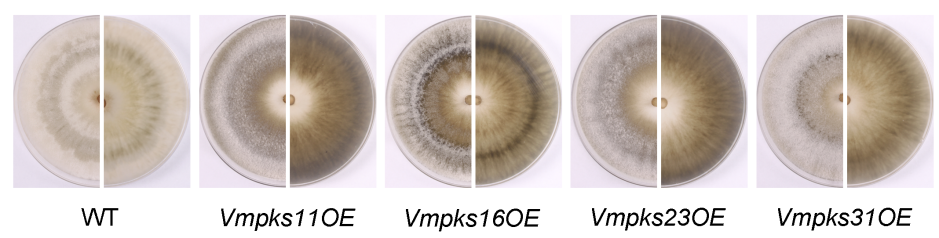
**

**Supplementary** **Figure 6. Pigment accumulation in** ***Vmpks11OE*, *Vmpks16OE*, *Vmpks23OE* and *Vmpks31OE*.** WT and mutant strains were cultured on PDA medium for five days. The left side of each plate is the front of colony and the right side is the back of colony.


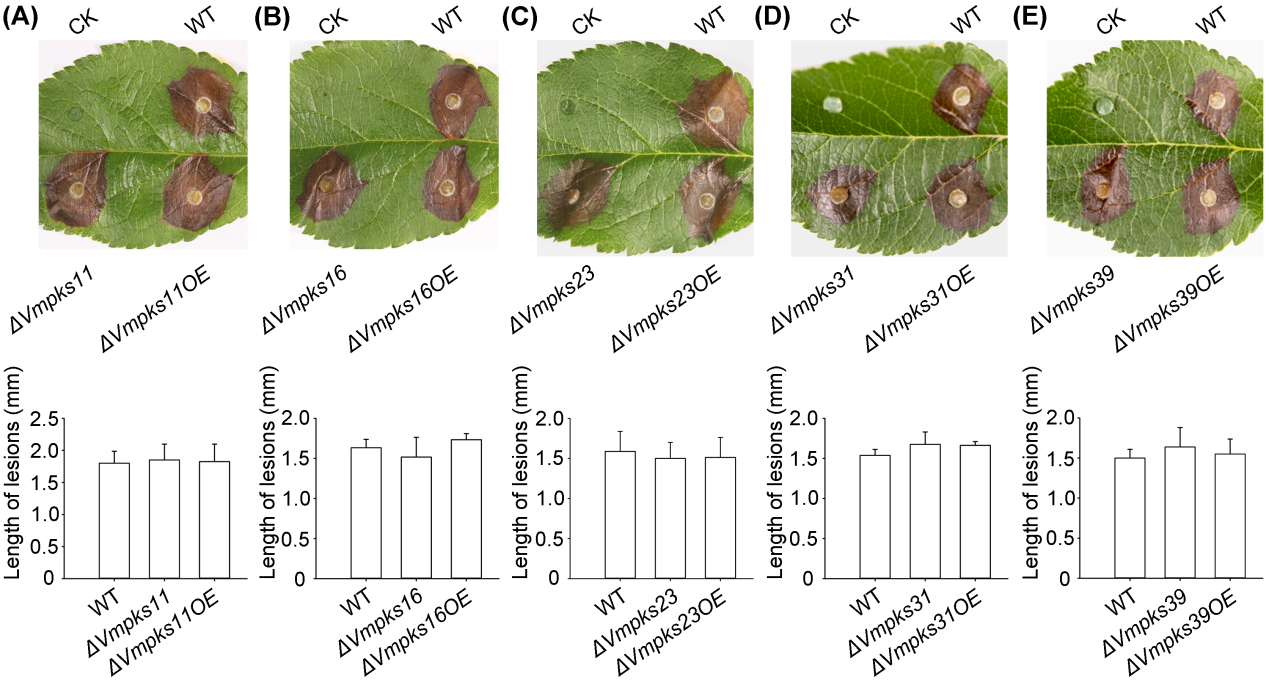


**Supplementary Figure 7. Pathogenicity tests of deletion and overexpression mutants of cluster *PKS11*, *PKS16*, *PKS23*, *PKS31* and *PKS39*.** WT and mutant strains of cluster *PKS11*, *PKS16*, *PKS23*, *PKS31* and *PKS39* were inoculated on detached leaves of *Malus domestica* borkh. cv. ‘Fuji’ and were cultured for three days. In bar graphs, means ± standard deviation (SD) of at least three biological replicates are shown. Significant differences would indicate with asterisks (*P*-value < 0.05).

**Supplementary**
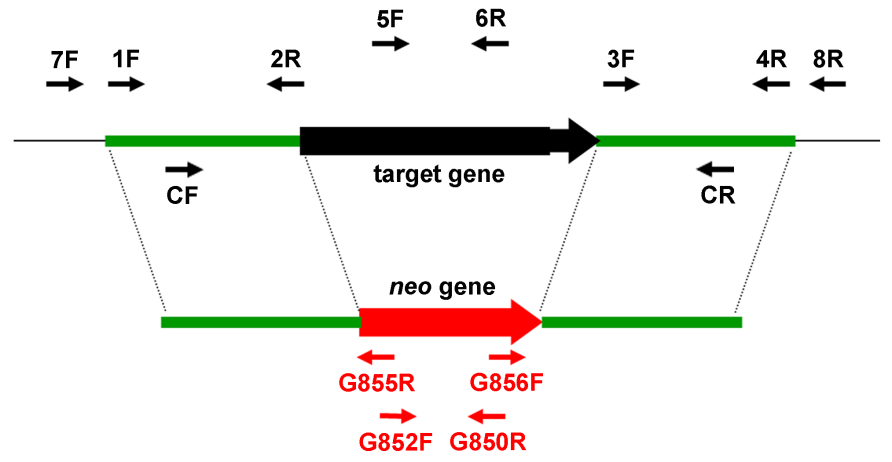
**Figure 8. The schematic representation of targeted gene replacement.** 1F, 2R, 3F, 4R, 5F, 6R, 7F, 8R, CF and CR are gene specific primers. Primer G855R, G856F, G852F and G850R are used for detecting fused DNA fragments and screening the knockout mutant of targeted genes.
