# Supplementary material for: LaeA Controls Virulence and Secondary Metabolism in Apple Canker Pathogen Valsa mali
Source: Front Microbiol. 2020 Nov 5;11:581203. doi: 10.3389/fmicb.2020.581203 (PMC7674932; doi:10.3389/fmicb.2020.581203)
Supplement: Supplementary file 3 [file Table_1.docx]

| **Type^a^** | **Down-regulated** | | **Up-regulated** | | **Description of the core enzyme** |
| --- | --- | --- | --- | --- | --- |
|  | **Cluster name^b^** | **Core enzyme Gene ID** | **Cluster name^c^** | **Core enzyme Gene ID** |  |
| PKS | *PKS5* | *VM1G_02156* | *PKS1* | *VM1G_00046* | Lovastatin diketide synthase |
|  | *PKS9* | *VM1G_02824* | *PKS21* | *VM1G_04822* |  |
|  | *PKS20* | *VM1G_04801* | *PKS36* | *VM1G_08248* |  |
|  | *PKS34* | *VM1G_08019* | ***PKS39*** | *VM1G_09018* |  |
|  |  |  | *PKS45* | *VM1G_11041* |  |
|  | ***PKS7*** | *VM1G_02489* | *PKS13* | *VM1G_03186* | Polyketide synthase |
|  | ***PKS11*** | *VM1G_03093* |  |  |  |
|  | *PKS15* | *VM1G_03589* |  |  |  |
|  | ***PKS16*** | *VM1G_03769* |  |  |  |
|  | ***PKS23*** | *VM1G_05383* |  |  |  |
|  | ***PKS31*** | *VM1G_07355* |  |  |  |
|  | *PKS17* | *VM1G_04331* | *PKS41* | *VM1G_09624* | Conidial yellow pigment biosynthesis polyketide synthase |
|  | *PKS22* | *VM1G_04961* |  |  |  |
|  | *PKS49* | *VM1G_02661* |  |  |  |
| NRPS | *NRPS4* | *VM1G_01528* |  |  | Linear gramicidin synthase |
|  | *NRPS47* | *VM1G_11144* |  |  | Acyl-CoA synthetase family member 2, mitochondrial |
|  | *NRPS10* | *VM1G_03054* | *NRPS27* | *VM1G_06774* | Nonribosomal peptide synthetase |
|  |  |  | *NRPS25* | *VM1G_06308* | Putative peroxisomal-coenzyme A synthetase |
|  |  |  | *NRPS52* | *VM1G_08051* | Oxygen-dependent choline dehydrogenase |
|  |  |  | ***NRPS14*** | *VM1G_03342* | Nonribosomal peptide synthetase like |
|  |  |  | *NRPS24* | *VM1G_05435* |  |
| NRPS/PKS |  |  | *NRPS/PKS3* | *VM1G_01285* | Polyketide synthase-nonribosomal peptide synthetase |
|  |  |  | ***NRPS/PKS33*** | *VM1G_07481* |  |
| TS | *TS60* | *VM1G_10824* |  |  | Dammaradiene synthase |

**Table 1 Down-regulated and up-regulated secondary metabolite biosynthetic gene clusters (SMBGCs) in the Δ*VmlaeA* mutant.**

^a^ PKS: polyketide synthesis; NRPS: non-ribosomal peptide synthesis; NRPS/PKS: hybrid non-ribosomal peptide and polyketide synthesis; TS: terpene synthesis.

^b^ Clusters marked in red are down -regulated in *ΔVmlaeA* at both transcriptional and translational level. Other clusters are down-regulated only at the transcriptional level.

^c^ Clusters marked in red are up-regulated in *ΔVmlaeA* at both transcriptional and translational level. Other clusters are up-regulated only at the transcriptional level.
