# Supplementary figures and images for: LaeA Controls Virulence and Secondary Metabolism in Apple Canker Pathogen Valsa mali
Source: Front Microbiol. 2020 Nov 5;11:581203. doi: 10.3389/fmicb.2020.581203 (PMC7674932; doi:10.3389/fmicb.2020.581203)

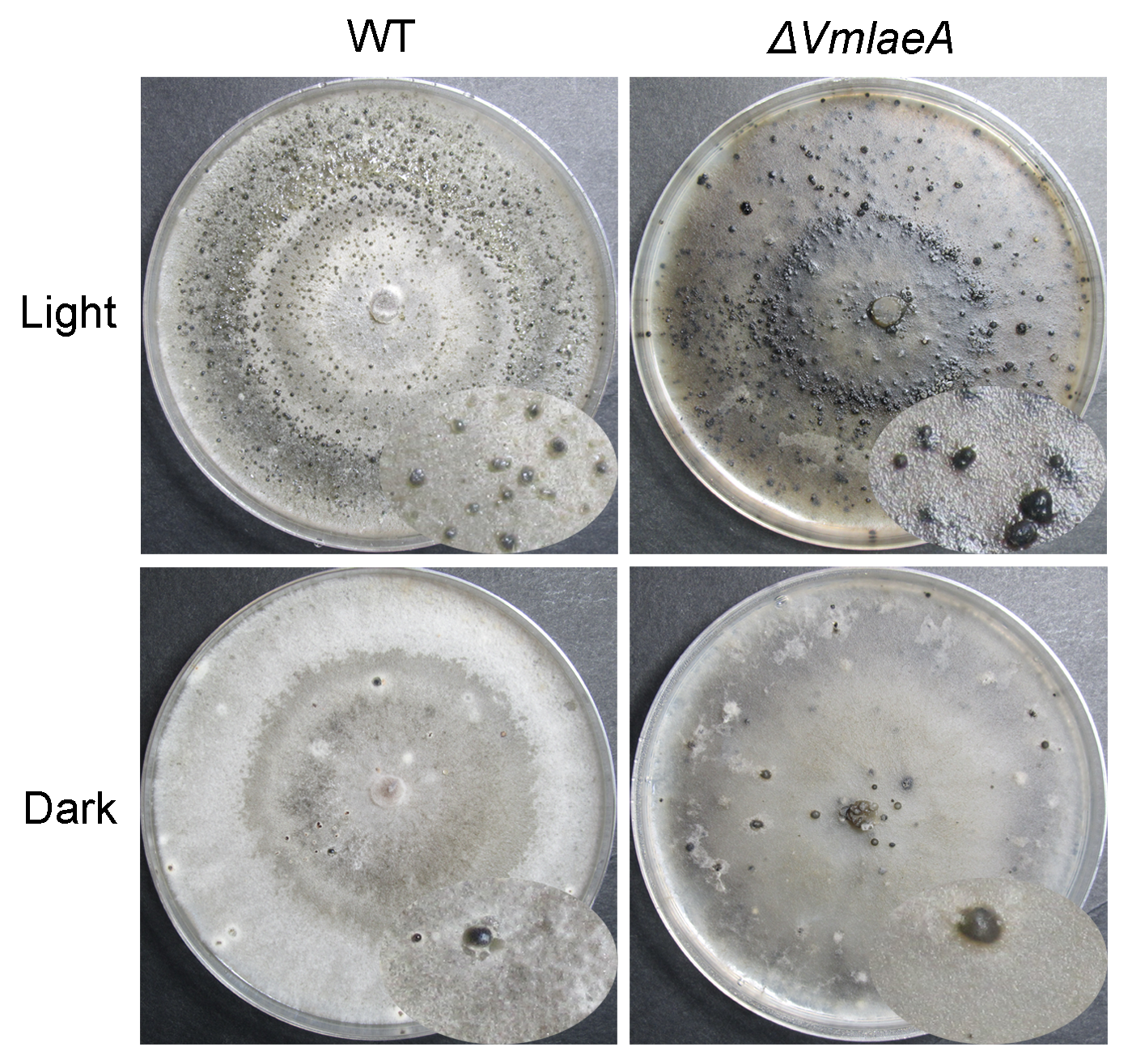

Supplement: Supplementary file 4 [file Image_1.TIF]

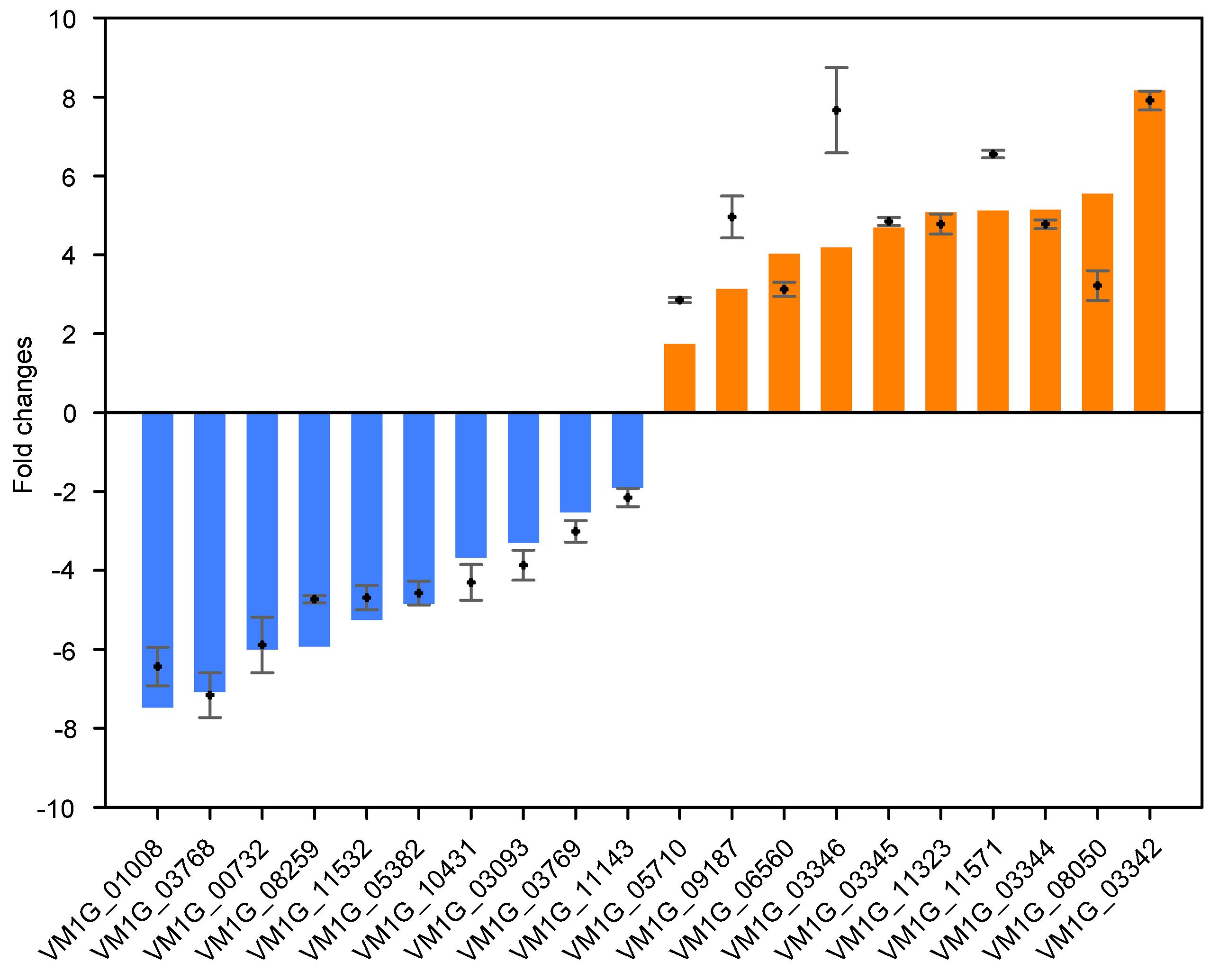

Supplement: Supplementary file 5 [file Image_2.TIF]

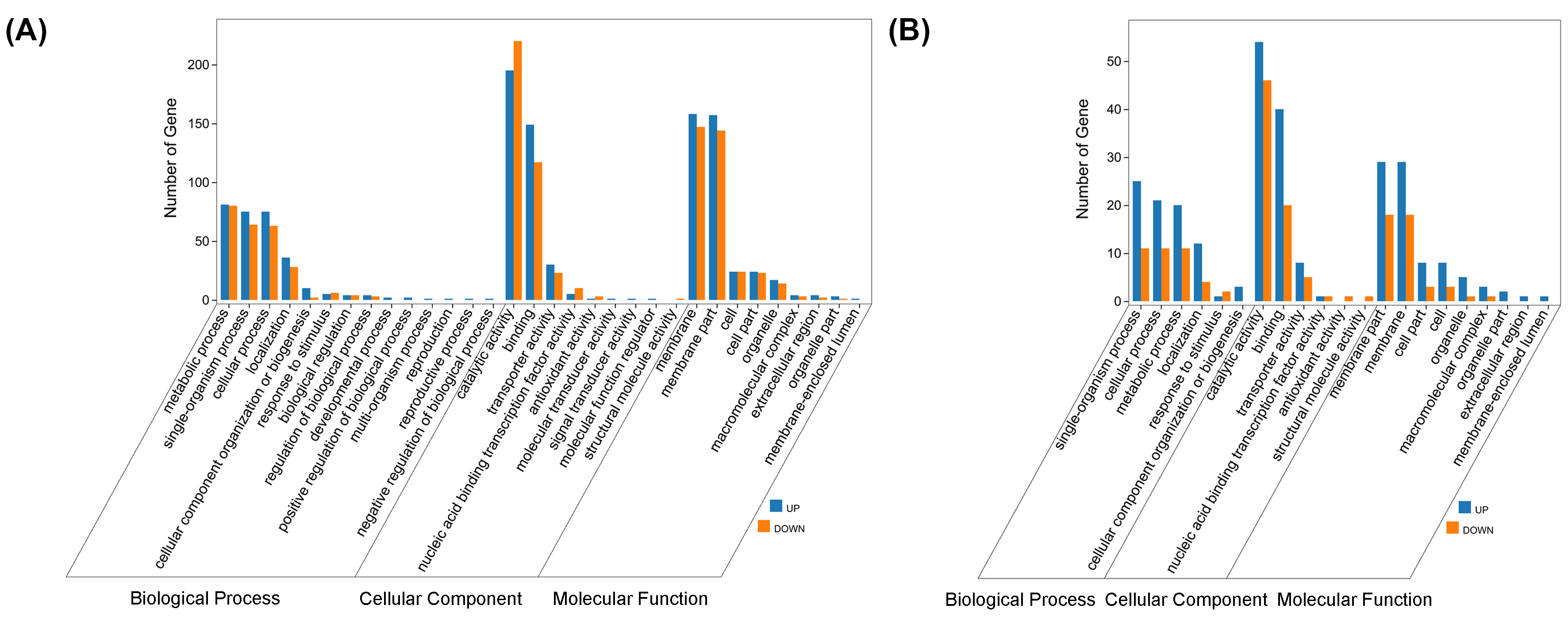

Supplement: Supplementary file 6 [file Image_3.TIF]

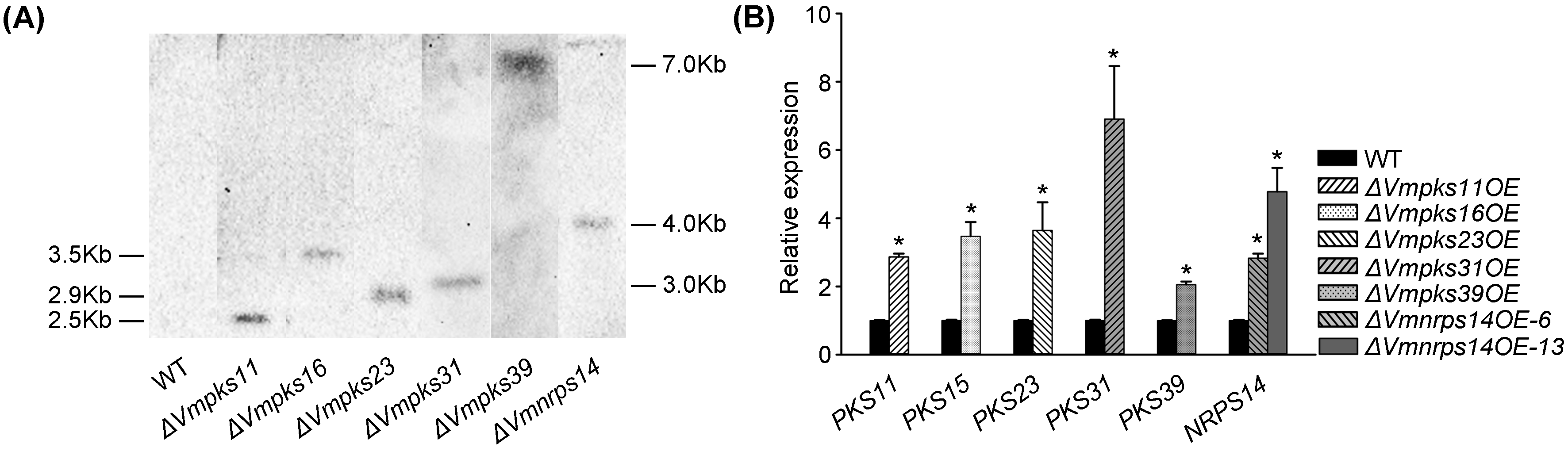

Supplement: Supplementary file 7 [file Image_4.TIF]

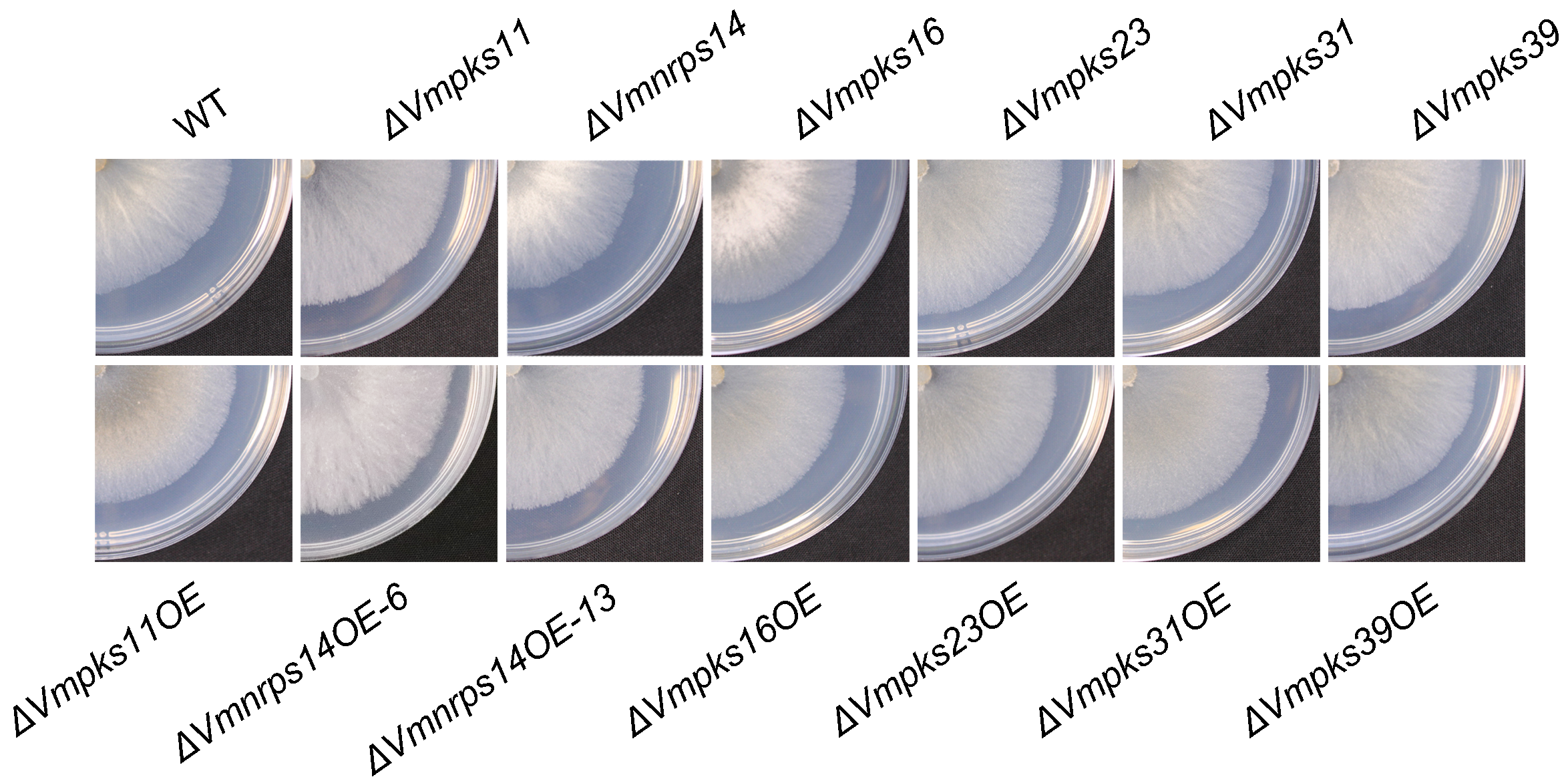

Supplement: Supplementary file 8 [file Image_5.TIF]

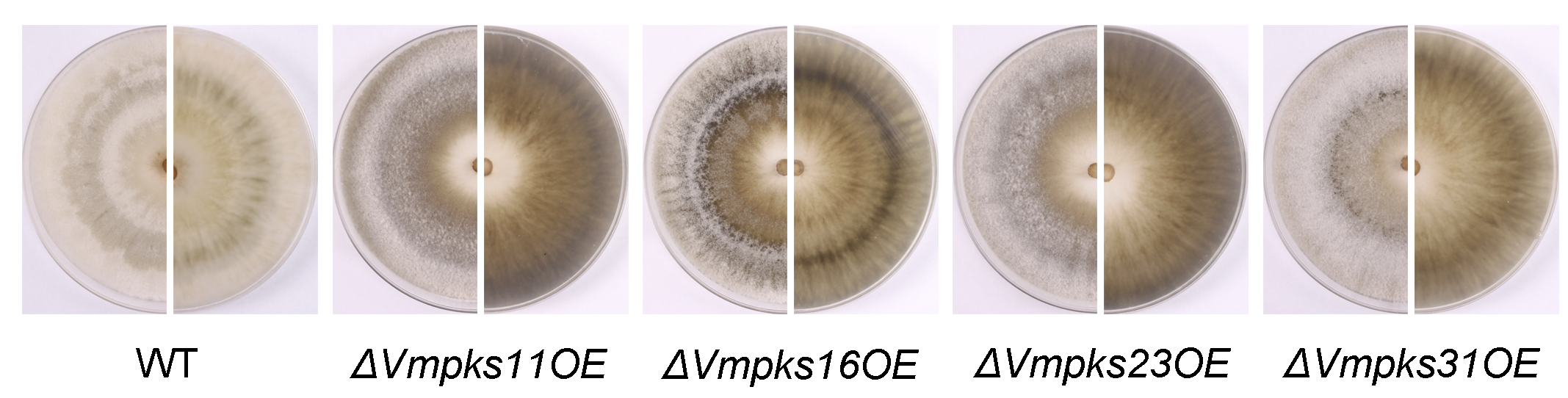

Supplement: Supplementary file 9 [file Image_6.TIF]

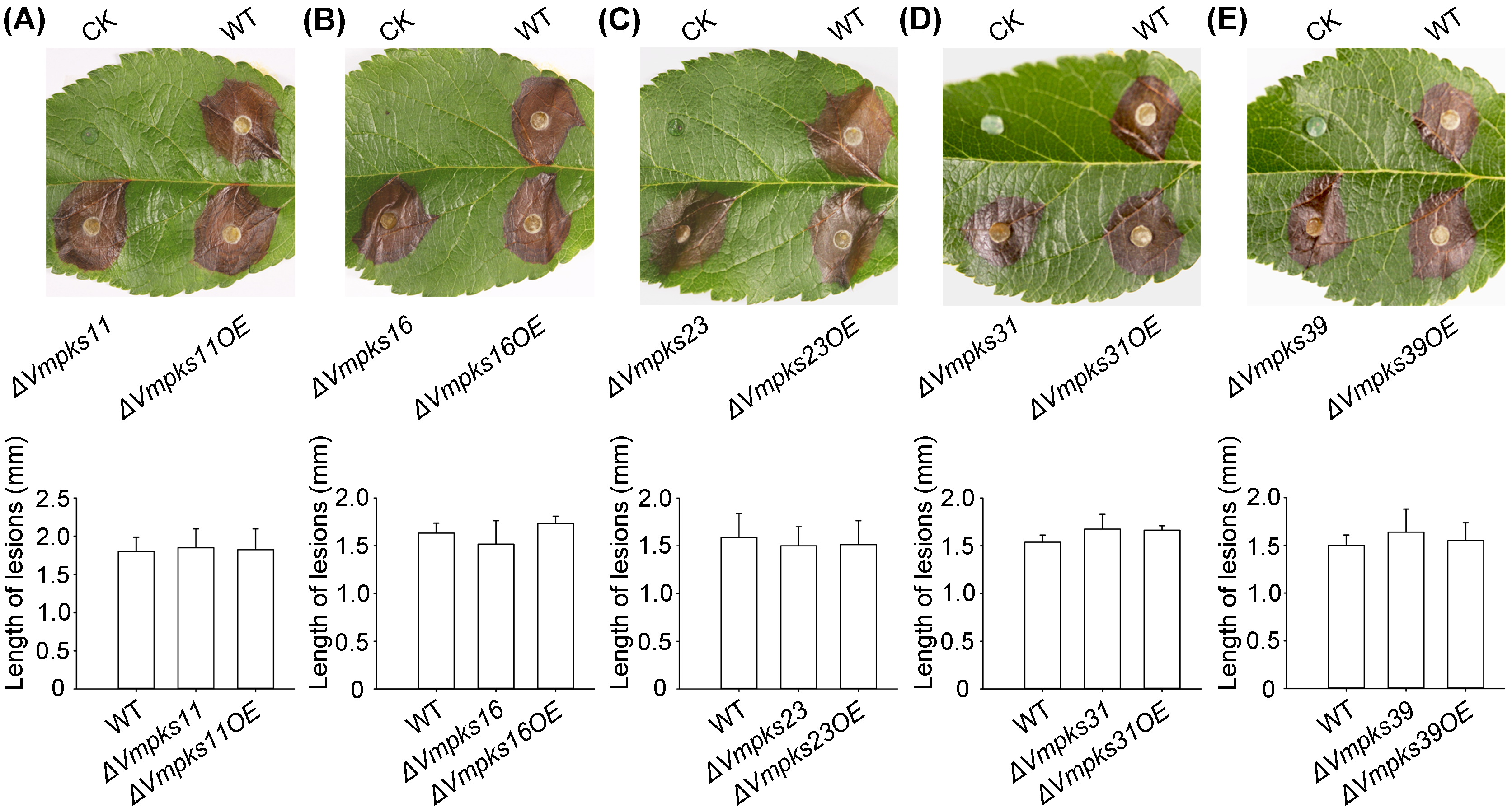

Supplement: Supplementary file 10 [file Image_7.TIF]

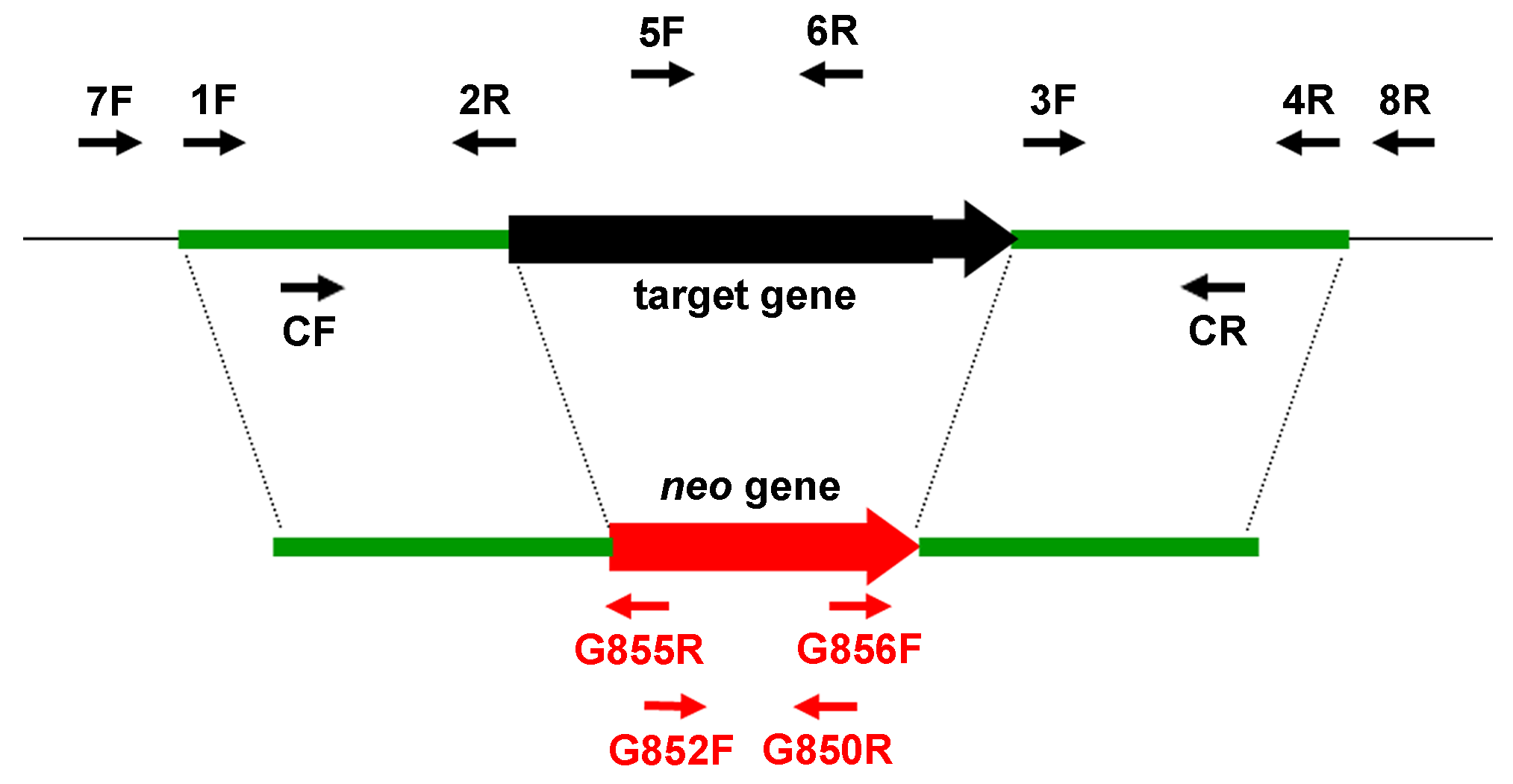

Supplement: Supplementary file 11 [file Image_8.TIF]
